# Enhanced release of primary signals may render intercellular signalling ineffective due to spatial aspects

**DOI:** 10.1038/srep33214

**Published:** 2016-09-20

**Authors:** Pavel Kundrát, Werner Friedland

**Affiliations:** 1 Institute of Radiation Protection, Department of Radiation Sciences, Helmholtz Zentrum München-German Research Center for Environmental Health (GmbH), Neuherberg, Germany

## Abstract

Detailed mechanistic modelling has been performed of the intercellular signalling cascade between precancerous cells and their normal neighbours that leads to a selective removal of the precancerous cells by apoptosis. Two interconnected signalling pathways that were identified experimentally have been modelled, explicitly accounting for temporal and spatial effects. The model predicts highly non-linear behaviour of the signalling. Importantly, under certain conditions, enhanced release of primary signals by precancerous cells renders the signalling ineffective. This counter-intuitive behaviour arises due to spatial aspects of the underlying signalling scheme: Increased primary signalling by precancerous cells does, upon reaction with factors derived from normal cells, produce higher yields of apoptosis-triggering molecules. However, the apoptosis-triggering signals are formed farther from the precancerous cells, so that these are attacked less efficiently. Spatial effects thus may represent a novel analogue of negative feedback mechanisms.

Individual cells rarely act as independent entities; their behaviour and responses to various stressors are closely coordinated within tissues and organs. Intercellular communication through gap junctions or diffusible signals enables keeping the balanced state of homeostasis; its malfunctioning underlies a number of diseases including cancer. Intercellular signalling and subsequent intracellular signal transduction and execution pathways possess complex characteristics. Due to positive and negative feedbacks, responses to signalling are typically not directly proportional to signal levels but exhibit non-linear features: low signal levels are often ineffective and the responses saturate at high signal levels. Many signalling processes including cell fate decisions, such as whether to divide or not, actually possess a binary, switch-like nature (reviewed e.g. in ref. 1).

Molecular mechanisms for a large number of signalling pathways have been identified. Systems biology approaches that use mathematical modelling as a useful complement to experimental research help understand the detailed behaviour of the pathways and the roles of the key players involved. The models vary as much as the studied processes do: Animal coat and other pattern formation can be represented successfully by reaction-diffusion models based on partial differential equations that explicitly account for spatial and temporal dependences of the underlying signalling and the feedback mechanisms^2^,^3^. Models of intracellular signal transduction pathways, e.g. the models for transforming growth factor type β (TGF-β) pathway^4^,^5^,^6^, typically do not treat spatial profiles in full detail but compartmentalize the region of interest. These models work with mean concentration levels of individual players such as TGF-β outside the cell, its receptors in cell membrane, and downstream signals such as R-SMAD and Co-SMAD in cytoplasm and in nucleus; their kinetics is described by ordinary differential equations. Recently, the potential importance of spatial effects beyond this compartmentalization has been highlighted^7^. Bystander effects, in which not only cells directly affected by a stressor (e.g. radiation impact) but also their neighbours respond to this stress, involve signalling processes of largely unknown nature. Nevertheless, in models of bystander effects, a single signal responsible for the studied process is commonly assumed^8^. The extent to which spatial and temporal aspects are considered differs substantially among the proposed models, cf.^8^,^9^,^10^ and references therein.

In this work, intercellular signalling is studied between precancerous, oncogenically transformed cells and the neighbouring normal cells. Oncogenically transformed cells possess some of the hallmarks of multistep carcinogenesis, such as changes in morphology, loss of contact inhibition, growth independent from specific growth factors, oncogene activation and tumour suppressor gene inactivation^11^,^12^,^13^,^14^,^15^. By transplantation of transformed cells into immunosuppressed animals, tumour formation can be induced; however, cells later extracted from these tumours possess additional functional changes compared to the originally injected transformed cells^15^. Thus, oncogenically transformed cells represent an *in vitro* system that largely mimics early-stage carcinogenesis^15^; they ‘reflect the cell culture equivalent of initiation’ in carcinogenesis^16^.

Another hallmark of transformed cells is their constitutive production of superoxide (O_2_^−·^) through membrane-based NADPH (nicotinamide adenine dinucleotide phosphate) oxidase^15^,^17^,^18^; superoxide plays a key role in maintaining the transformed state of the cells and in controlling their proliferation^19^. On the other hand, superoxide is also critically involved in intercellular signalling between precancerous cells and their normal neighbours, upon which the precancerous cells are selectively removed by apoptosis (so-called intercellular induction of apoptosis, IIA). The mechanism that underlies IIA was identified experimentally^15^,^18^ and is outlined in Fig. 1: Through the release of TGF-β and/or activation of its latent form, transformed cells trigger the release of nitric oxide (NO^·^) and peroxidase (POD) in neighbouring (transformed or normal) cells. O_2_^−·^, NO^·^ and POD (‘primary signals’) undergo a biochemical reaction cascade with two interconnected pathways: (i) peroxidase/hypochlorous acid pathway, closely related to signalling that underpins the immune defence against bacteria via phagocytosis by neutrophils^20^, and (ii) nitric oxide/peroxynitrite pathway, which involves species also implicated in host defence or in a wide variety of signalling systems including those regulating blood flow^21^. The IIA signalling cascade is depicted in detail in Fig. 1b; the main involved reactions are listed in Table 1. This signalling eventually leads to the formation of hydroxyl radicals (^·^OH) that induce peroxidative modifications to membrane lipids. Through an intracellular signal transduction pathway, this membrane damage may trigger the execution of apoptosis. Due to the short range of superoxide, hydroxyl radicals are formed predominantly in the close vicinity of the superoxide-producing transformed cells; therefore IIA works selectively against the transformed phenotype.

The IIA phenomenon has been suggested to serve as a natural anti-carcinogenic mechanism^15^,^11^; previous modelling has demonstrated that IIA may limit the growth of a population of transformed cells and induce a kind of dormancy^22^. *In vitro* experiments have shown that the IIA mechanism is effective against cells transformed by viruses, oncogene activation, irradiation or spontaneous transformation of diverse cells, including rodent and human epithelial, endothelial, haematopoietic and fibroblast cells. Likewise, a large variety of rodent and human cells can serve as effector cells, i.e. release sufficient amounts of signals that are needed for making the IIA effective^15^. Depending on the cell lines, cell densities and other conditions of the system, the peroxidase or the peroxynitrite pathway may dominate, or both may contribute comparably^15^,^23^. In addition, two further IIA pathways have been identified experimentally, but these play only minor roles^18^, and hence they are not considered throughout this paper. The phenomenon of IIA has been studied systematically *in vitro* with transformed and normal cells grown on the same dish, as well as using co-culture experiments in which the two cultures were physically separated by a distance of about 1 mm but shared the growth medium^15^,^18^.

In this paper, the previously published mathematical model of IIA^22^, which was originally limited to the peroxidase/hypochlorous acid pathway, has been extended to account for both major IIA pathways and their interconnection. Detailed time- and space-resolved numerical simulations of the system have been performed, and their results have been approximated by analytical and iterative procedures. Surprising behaviour of the system is revealed: With increasing release of primary signals, the effect may not only increase and saturate but, paradoxically at first sight, also decrease; less can be more. This counter-intuitive, inverse behaviour arises due to spatial effects: Increased release rates of primary signals (O_2_^−·^) by precancerous cells do produce, upon reaction with normal cell-derived factors (NO^·^), higher total yields of signalling molecules (ONOO^−^ and subsequently its decay product ^·^OH) that can trigger apoptosis. However, these apoptosis-triggering signals are formed farther away from the precancerous cells, which are hence attacked and triggered for apoptosis less efficiently. To our knowledge such inverse behaviour due to spatial aspects has not been identified yet in systems biology of signalling systems, and significantly extends the set of their known non-linear features.

## Results

### Removal of transformed cells by apoptosis

The mechanistic model of IIA with parameters listed in Tables 2 and 3 correctly reproduces the observed response^24^ of transformed fibroblasts to externally added donors of signalling species (Fig. 2a). As shown in Fig. 2b, the modelled induction of apoptosis in transformed 208F src3 rat fibroblasts co-cultured with non-transformed 208F fibroblasts is consistent with the measured data^25^ too, with both pathways present (red points and line) as well as with the nitric oxide/peroxynitrite pathway inhibited (green points and line). The dashed lines exemplify the uncertainty of parameter determination; they correspond to an alternative parameter set consistent with the data (set A in Tables 2 and 3). In Fig. 2c,d, model calculations are compared to data with the same cell lines obtained in a different institute^26^. Contrary to Fig. 2b, in these experiments eventually all cells undergo apoptosis. To account for this biological variability, a minimally adjusted parameter set (set B in Tables 2 and 3) has been used; in particular, the repair of the induced damage has been neglected. While the reported calculations overestimate the extent of autocrine destruction in a population of transformed cells (Fig. 2c) at high cell density, its kinetics is reproduced correctly (Fig. 2d, magenta points and line). A reasonable agreement is obtained also for the kinetics of apoptosis in transformed cells co-cultured with their non-transformed parental cell line (Fig. 2d, red, green and blue points and lines). An even higher degree of conformity with the particular data can be obtained if individual data sets are modelled independently (as illustrated by the dashed blue line in Fig. 2d; parameter set C in Tables 2 and 3).

### General patterns of system behaviour

The model works with mechanistically distinct parameters. However, not all the parameters could have been identified from the available data; e.g. it is not possible to determine the release rates of species in absolute terms since their concentrations have not been measured directly^22^. The biological variability of IIA signalling is actually much higher than what is illustrated above. In some cell lines the peroxidase/HOCl pathway dominates, while others rely solely on the nitric oxide/peroxynitrite pathway, and yet others signal through both pathways to a comparable extent^23^. While the underlying signalling scheme is valid generally, the biologically relevant parameters may differ considerably from those reported in Tables 2 and 3, derived for rat fibroblasts. Therefore, to assess the general behaviour of the IIA signalling system, a systematic study has been performed on the roles of individual model parameters and their influences upon the dynamics of the growth of a transformed cell population. Single model parameters have been varied over wide ranges of potentially biologically relevant values, for simplicity keeping the other parameters at their ‘standard values’ from Tables 2 and 3; typically the parameters have been allowed to vary by 3–6 orders of magnitude. Numerical simulations of IIA signalling and of the resulting dynamics of the transformed population have been complemented by relatively simple analytical and more accurate iterative approximations.

The results of these simulations show that basic cell biology parameters such as the doubling time or confluence density affect the kinetics of apoptosis in the anticipated way. The same holds for model parameters that describe cellular response to the induced membrane damage. The results are given in Supplementary Material.

In the following, the effectiveness of IIA signalling is assessed in terms of the yields of hydroxyl radicals (^·^OH) in the close vicinity of transformed cells; note that only the signalling step is included in this concept, not the subsequent cellular response. As expected, increasing release rates or lifetimes of the involved signalling species generally increase the IIA signalling effectiveness, typically in a sigmoidal manner: Increasing the release rate or lifetime of a species has no effect initially if another pathway dominates, makes the overall signalling more effective in the region where the particular pathway is active, and finally the signalling effectiveness saturates when the species under study is no longer limiting for the given pathway. This is illustrated for the release of NO^·^ by transformed cells in Fig. 3a: For the standard parameters, enhancing NO^·^ release rates up to about 10^−19^ mol/s per transformed cell does not alter the signalling outcome (Fig. 3a); the majority of superoxide undergoes spontaneous dismutation, and the HOCl pathway (red line) dominates the signalling in this region. When the NO^·^ release rates are further enhanced, reaction of superoxide with NO^·^ outcompetes its spontaneous dismutation, and the IIA signalling effectiveness rapidly grows, about proportionally to the NO^·^ release rate. The signalling is dominated by the peroxynitrite pathway in its autocrine mode (black line), where both O_2_^−·^ and NO^·^ are released by transformed cells. Above NO^·^ release of 10^−16^ mol/s per transformed cell, the peroxynitrite pathway is no longer limited by NO^·^ but by O_2_^−·^ (given the 1:1 stoichiometry of their reaction), and hence further increasing the NO^·^ release rate no longer modulates the signalling effectiveness. The peroxynitrite pathway with NO^·^ derived from effector cells (inter-culture mode, green line) does not play an important role in this case. Note that the results of detailed numerical simulations of the IIA signalling (squares) are perfectly reproduced by the iterative approach (blue line); also the relatively simple analytical formulas work fine (dashed blue line). Similar sigmoidal behaviour is obtained for varying the release rate of NO^·^ by effector cells, the amount of peroxidase present, or the lifetimes of NO^·^, HOCl, H_2_O_2_ or ONOO^−^ (Supplementary Material).

The signalling effectiveness, however, depends non-trivially on the release rate of O_2_^−·^ by transformed cells (Fig. 3b). At high release rates of O_2_^−·^, above 10^−16^ mol/s per transformed cell for the standard parameters from Tables 2 and 3, the signalling is dominated by the HOCl pathway (red line). Its effectiveness rapidly increases, with up to third power of the release rate of superoxide, cf. analytical formulas in Supplementary Material. Also below 10^−18^ mol/s per transformed cell, the signalling effectiveness increases with O_2_^−·^ release rate; here the peroxynitrite pathway dominates, but is limited by the production of O_2_^−·^, and hence the signalling effectiveness increases proportionally to its release rate (cf. analytical formulas in Supplementary Material). In the region of intermediate release rates of O_2_^−·^ by transformed cells, however, the overall signalling effectiveness is predicted to *decrease* with increasing releases of O_2_^−·^. Here, the inter-culture mode of the peroxynitrite pathway (O_2_^−·^ produced by transformed and NO^·^ by normal cells; Fig. 3b, green line) plays an important role, and its effectiveness decreases with increasing releases of O_2_^−·^. The reason is as follows: O_2_^−·^ possesses a short lifetime and hence a limited diffusion length, so that its concentration rapidly decreases with increasing distance from the transformed cells. The reaction of O_2_^−·^ with NO^·^ is effective only in a relatively small region where their concentrations are comparable. When more superoxide is produced by the transformed cells, this region gets shifted farther from the transformed cells (cf. Figure S2 in Supplementary Material). Although more peroxynitrite is formed in absolute terms there, actually less peroxynitrite diffuses back to the transformed cells due to its low stability compared to NO^·^ (Table 3). This then reduces the signalling effectiveness of this pathway. Note that the results of full numerical simulations (squares) are correctly reproduced by the iterative approach (solid blue line) over all three regions just discussed. The analytical formulas (dashed blue line) provide correct trends but underestimate signalling effectiveness at the highest release rates of superoxide, due to having overestimated the consumption of O_2_^−·^ in its reaction with HOCl (cf. Supplementary Material). More importantly, the analytical formulas overestimate the signalling at low release rates of O_2_^−·^, because the competition for superoxide between NO^·^ released by transformed and effector cells is neglected, i.e. each O_2_^−·^ molecule is used twice by the analytical formulas in this region. Nevertheless, the analytical formulas do capture the predicted decrease in signalling effectiveness of the peroxynitrite pathway in the intermediate region of O_2_^−·^ release rates, and can serve as a quick tool to roughly quantify and analyse this trend. Also note that shifting the effective source of peroxynitrite farther away from the population of transformed cells enhances the levels of peroxynitrite diffusing to the population of normal cells (cf. Supplementary Material, Figure S2); if the resulting ^·^OH attacks to normal cells were sufficient to trigger their apoptosis, this effect would reduce the selectivity of IIA towards the transformed phenotype.

The lifetime of superoxide is also predicted to yield a non-trivial influence upon the signalling effectiveness (Fig. 3c). Again, the decreasing part at short lifetimes follows from the properties of the peroxynitrite pathway in its inter-culture mode (green line). At long lifetimes of superoxide, the iterative approach (solid blue line) and even more the analytical formulas (dashed blue line) become inaccurate, as the underlying approximation of mutual reactions by local absorption terms loses its validity. Note that the concept of superoxide lifetime used here accounts for the removal of superoxide from the reaction cascade, while most antioxidants convert this species to hydrogen peroxide; the related simulations are presented in Supplementary Material.

### Signalling predicted effective for low or high but not medium densities of transformed cells challenged by high densities of normal cells

The predicted complex dependence of the IIA signalling effectiveness on the release rate of superoxide can be translated into predictions that could be directly tested experimentally. The inverse behaviour of the signalling, namely signalling effectiveness that decreases with increasing production of primary signals (superoxide) by transformed cells, is predicted to happen under conditions when the inter-culture mode of the peroxynitrite pathway dominates and decreases. This means rather small densities of transformed cells, so that the HOCl pathway is not dominant, but not too small as otherwise the production of superoxide were limiting. At the same time, high densities of normal cells are needed, providing high amounts of nitric oxide. Indeed, at high seeding densities of effector cells, the IIA model predicts the percentage of transformed cells undergoing apoptosis to show a bell-shaped (U-shaped) dependence on the seeding density of transformed cells, as shown for 24 h co-culture in Fig. 4a. The decreasing part of this dependence, present at densities of transformed cells below about 20 cells/mm^2^, is due to the peroxynitrite pathway in its inter-culture mode. The rapid increase in signalling efficiency at densities of transformed cells above 100 cells/mm^2^ follows from the increasing activity of the HOCl pathway; reducing the effectiveness of the HOCl pathway by selective inhibitors such as taurine, the model predicts only the decreasing ramp of this U-shaped curve to remain present (not shown). The predicted effects get even more pronounced if higher cell densities are achievable; e.g. for the parameter set A that works with higher confluence densities, sharper and narrower U-shaped curves for apoptosis in dependence on transformed cell density are predicted (Fig. 4b). On the contrary, if confluence densities were lower than the standardly assumed ones, the inverse behaviour would be less pronounced (not shown).

## Discussion

The given mathematical model was previously shown to correctly represent the induction of apoptosis through the peroxidase/HOCl pathway^22^. In this work the modelling has been extended to the second major pathway, nitric oxide/peroxynitrite pathway, and the interplay of the two pathways. To assess characteristic patterns of system behaviour, detailed numerical simulations of the IIA signalling have been performed. Individual model parameters have been varied over several orders of magnitude. An iterative approach has been proposed that reproduces the results of full numerical simulations and enables systematic modelling studies of the population dynamics of transformed cells. Analytical formulas have been developed that provide an even simpler and quicker tool but do not reach the accuracy of the iterative procedure (cf. Fig. 3b,c).

In some experiments, IIA was studied in transformed cells seeded in clumps directly onto a dish with normal cells. For this case, the one-dimensional approximation used here likely reflect the general features of the system, but realistic simulations may need to be extended to a three-dimensional picture that would account for the contributions to signalling from nearby and distant cells. This work is focused on co-culture experiments with transformed cells grown in wells and normal cells in porous inserts, so that the two cultures shared the growth medium but were physically separated from each other by a 1 mm distance; the one-dimensional approximation is justified then.

The agreement of the iterative results with full numerical simulations indicates the validity of the assumption that mutual reactions between signalling species can be approximated by local absorption terms. The results show that this approximation works not only for the almost diffusion-limited reaction of superoxide with nitric oxide (with reaction rate constant *k* = 6 × 10^9^ M^−1^ s^−1^), but also for the much slower dismutation of superoxide (*k* = 2 × 10^5^ M^−1^ s^−1^), and could be used in reaction systems in other contexts too. In particular, this and other model results may have direct implications for models of the antimicrobial activity of neutrophils, given the similarities in the underlying signalling.

The IIA model works with parameters that are mechanistically distinct and, as such, in principle determinable if sufficient data on IIA were available. However, although e.g. the cellular release rates of superoxide and nitric oxide have been determined in other biological systems^27^, no such direct measurements have been performed for the IIA signalling. The parameter values used in this work (Tables 2 and 3) are estimates derived previously from the induction of apoptosis in transformed rat fibroblasts by defined amounts of signals added externally as well as upon co-culture with normal rat fibroblasts^22^,^28^. However, given the limited data available, not all model parameters could have been determined simultaneously. The reported values of ^·^OH concentrations, for instance, cannot be taken as absolute predictions of the model, but are related to cellular sensitivity to membrane damage. This parameter gives the amount of peroxidative damage to membrane lipids (more precisely, its initiation events) at which the cells typically start undergoing apoptosis; the values given in Table 2 correspond to about 1% of membrane lipids being attacked. However, the same apoptosis induction would result if both this sensitivity parameter and the levels of ^·^OH were e.g. 10-times lower. The reported systematic simulations and the ability to approximate their results by the iterative approach make the impossibility to estimate the values of specific model parameters less critical, since they enable drawing general conclusions on the properties of the given signalling system.

In particular, the present results predict that the outcome of the given intercellular signalling may, paradoxically at first sight, decrease with an increasing release of primary signals, superoxide, by transformed cells. It would be very challenging if not impossible to test these predictions directly, as this would require experiments in which the production of superoxide by transformed cells were manipulated in a controlled way without affecting their other properties. However, what matters is the total flux of superoxide from transformed cells, which is given by the per-cell release rate and cell density. Hence the given model prediction can be translated into another one that could be tested experimentally. Namely, this inverse behaviour is predicted under specific conditions:

(1) The signalling has to be dominated by the inter-culture mode of the peroxynitrite pathway, in which superoxide released by transformed cells reacts with nitric oxide derived from normal cells. Thus this signalling mode has to be more effective than the HOCl pathway, which is fulfilled for small densities of transformed cells (or if the lifetime of HOCl or POD levels were reduced). At the same time, the peroxynitrite pathway in its autocrine mode (i.e. with both superoxide and nitric oxide derived from transformed cells) has to be relatively weak too. This is granted in this work by the assumption that TGF-β pre-treated cells release a substantially higher amount of peroxidase and nitric oxide than untreated cells do (cf. Table 3); this assumption reflects the observation that TGF-β pre-treatment of effector cells markedly enhances the extent and rate of apoptosis induction^25^,^26^,^29^. However, even if normal cells released NO^·^ in amounts comparable to those derived from transformed cells, which might be expected for normal cells without TGF-β pre-treatment, the peroxynitrite pathway in its autocrine mode will be weaker than the inter-culture mode if small densities of transformed cells are used relative to those of normal cells.

(2) The effectiveness of the peroxynitrite pathway in its inter-culture mode needs to decrease with the increasing production of superoxide, i.e. be limited by the levels of NO^·^ from normal cells rather than by those of transformed-cell derived O_2_^−·^. Experiments with transformed cells exposed to high levels of NO^·^ from a chemical donor with and without a donor of superoxide have shown that the transformed cells alone do produce sufficient amounts of superoxide^24^. The second condition thus translates into the requirement that the density of transformed cells be not too small compared to that of normal cells.

(3) The diffusion distance of nitric oxide needs to exceed that of peroxynitrite. This is fulfilled, as the stability of NO^·^ is considerably higher than that of peroxynitrite, *in vitro* (Table 3) as well as *in vivo*^21^,^30^,^31^,^32^,^33^.

Although the predicted inverse behaviour appears counter-intuitive, it possesses a clear mechanistic interpretation. It arises as a consequence of a reaction between signals with spatially distinct sources, and of a relatively low stability of the reaction product: Enhancing the release of superoxide from transformed cells shifts the region to which the reaction is effectively confined farther away from the transformed cells. Higher amounts of reaction product, peroxynitrite, are formed there, but due to the shorter diffusion distance of peroxynitrite compared with the reactant, nitric oxide, only lower amounts of peroxynitrite diffuse towards the transformed cells and, consequently, these cells are exposed to fewer attacks of hydroxyl radicals. On the contrary, the amounts of peroxynitrite diffusing to normal cells get larger, which may reduce the selectivity of IIA towards the transformed phenotype.

For high densities of normal cells and moderate but not too low densities of transformed cells, the model thus predicts the IIA signalling effectiveness to decrease with increasing release of primary signals, superoxide, from transformed cells. As a consequence, bell-shaped curves of apoptosis are predicted in dependence on the density of transformed cells co-cultured with high densities of normal cells (Fig. 3). These predictions could be tested in dedicated experiments. Note that due to the biological variability and the impossibility to derive all parameter values from the available data, the predictions have to be seen as qualitative or semi-quantitative ones only, as the predicted bell-shaped curves may get shifted in cell densities and/or in time.

Interestingly, similar bell-shaped curves have already been reported in experiments on the induction of apoptosis in transformed cells with large amounts of nitric oxide provided by a chemical donor, in dependence on the concentrations of superoxide dismutase added^34^. Although nitric oxide was provided externally rather than by normal cells, the two scenarios bear large similarities: The amounts of nitric oxide were very high in the experiment; high levels of nitric oxide and hence high densities of normal cells are needed for the inverse behaviour of IIA signalling to be predicted. Superoxide dismutase modulates the levels of superoxide by catalysing its dismutation into hydrogen peroxide; varying the densities of transformed cells also modulates the concentration of superoxide. Although it is tempting to speculate that these similarities may lend additional confidence to the present predictions, definitely these should be tested directly by dedicated experiments.

The predicted complex behaviour of IIA signalling may have important implications for *in vivo* carcinogenesis too. Compared with the *in vitro* co-culture experiments, *in vivo* lifetimes of all species involved in IIA signalling are much shorter, cell densities are higher, and the effective distances between the transformed and normal populations are smaller. In early-stage carcinogenesis, a relatively small population of transformed cells (e.g. a small spheroid) is challenged by a huge amount of normal cells. Under such conditions, the model predicts (results will be published elsewhere) the HOCl pathway to be suppressed due to its supra-linear dependence on superoxide (cf. analytical formulas in Supplementary Material) and the peroxynitrite pathway, weakened too but to a smaller extent only, to dominate. Analogously to Fig. 3b, the peroxynitrite pathway’s effectiveness would increase with the size of the transformed population up to a certain limit where a critical flux of superoxide would be produced, and then the signalling effectiveness would decrease with further increasing population sizes. Reaching this critical size or enhancing the production of superoxide might thus represent an escape mechanism for transformed cells from the anti-carcinogenic control by IIA. Whether this effect really happens and how it is related to the experimentally demonstrated expression of catalase on the surface of tumour cells^18^,^29^ needs to be elucidated experimentally.

Low doses of ionizing radiation have been shown to modulate signalling by TGF-β as well as by reactive oxygen and/or nitrogen species, and to affect the extent and rate of apoptosis in transformed cells challenged by normal ones^15^,^35^. The complex properties of the underlying signalling predicted here may have important implications for carcinogenesis induced by low-dose radiation: In radiobiological experiments *in vitro*, low-dose irradiation enhances the release of superoxide from transformed cells and of peroxidase (and potentially also of nitric oxide) from both transformed and normal cells^15^. Consequently, an enhanced induction of apoptosis in transformed cells has been reported^35^. The modelling reproduces this observation, i.e. an enhancement of this anti-carcinogenic process by radiation *in vitro*. However, as discussed above, under *in vivo* conditions the model predicts the peroxynitrite pathway to dominate the signalling scheme. Enhanced release of superoxide from irradiated precancerous cells may then reduce the overall signalling outcome, so that the precancerous cells would be removed by apoptosis to a smaller extent only or even not at all. Radiation would then reduce the given anti-carcinogenic process, i.e. it would act pro-carcinogenically *in vivo*, as is well known from epidemiological studies. The model thus solves the discrepancy between *in vitro* radiobiological and *in vivo* epidemiological evidence by predicting that radiation typically enhances IIA *in vitro* but acts in the opposite direction *in vivo*; this issue will be discussed in detail elsewhere.

In summary, the present results predict that intercellular signalling may not only saturate, but even get less effective in absolute terms with increasing releases of primary signals. This counter-intuitive behaviour follows from a reaction between primary signals released from spatially distinct sources, transformed and normal cells in the signalling system studied here. To our knowledge, such inverse behaviour resulting from spatial effects has not been identified yet in intercellular signalling systems. The reported model predictions for the selective removal of transformed cells by apoptosis induced upon signalling by neighbouring normal cells likely affect the implications of this phenomenon in carcinogenesis and its modifications by low-dose radiation *in vivo*.

## Methods

### Mathematical model of intercellular signalling and induction of apoptosis

**Figure 1:**
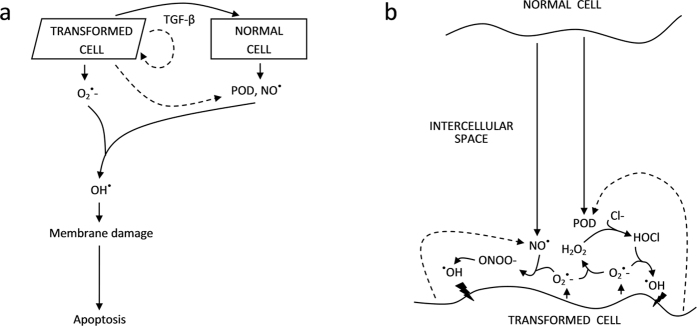
Induction of apoptosis in oncogenically transformed (precancerous) cells upon signalling by neighbour cells^15^,^18^. Panel a: Transformed cells constitutively produce superoxide (O_2_^−·^), which is involved in maintaining their transformed state and in controlling their proliferation. They also release transforming growth factor type β (TGF-β), which triggers neighbouring cells to release nitric oxide (NO^·^) and peroxidase (POD). TGF-β may affect transformed cells themselves to release these signals too (dashed lines). Through a cascade of biochemical reactions, hydroxyl radicals (^·^OH) are formed. These induce peroxidative damage to lipids in cell membrane, which then triggers intracellular pathways leading to apoptosis. Panel b: Detailed view of the intercellular signalling cascade, showing the two major pathways identified experimentally, the nitric oxide/peroxynitrite and peroxidase/hypochlorous acid pathways. In the peroxidase pathway, transformed cell-derived O_2_^−·^ dismutates into hydrogen peroxide (H_2_O_2_), which is by POD converted in part to water and in part, using abundant chlorine anions (Cl^−^), to hypochlorous acid (HOCl). Upon reaction of HOCl with O_2_^−·^, hydroxyl radicals are formed. In the peroxynitrite pathway, O_2_^−·^ reacts with NO^·^ to form peroxynitrite anion (ONOO^−^), whose conjugate acid (ONOOH) decays into NO_2_^·^ and ^·^OH. Two modes of the peroxynitrite pathway can be distinguished, the autocrine mode with NO^·^ derived from transformed cells themselves (dashed arrow) and the inter-culture mode with NO^·^ provided by normal cells (solid arrow). Due to the short lifetime and hence a small diffusion distance of O_2_^−·^, all reactions occur predominantly in the vicinity of transformed cells, and the induction of apoptosis is highly selective to the transformed phenotype. Tumour cells produce superoxide too, but in addition also express catalase (not depicted) that abrogates the signalling and makes tumour cells resistant to the intercellular induction of apoptosis^18^,^29^. Additional details on the signalling can be found in ref. 18.

**Figure 2:**
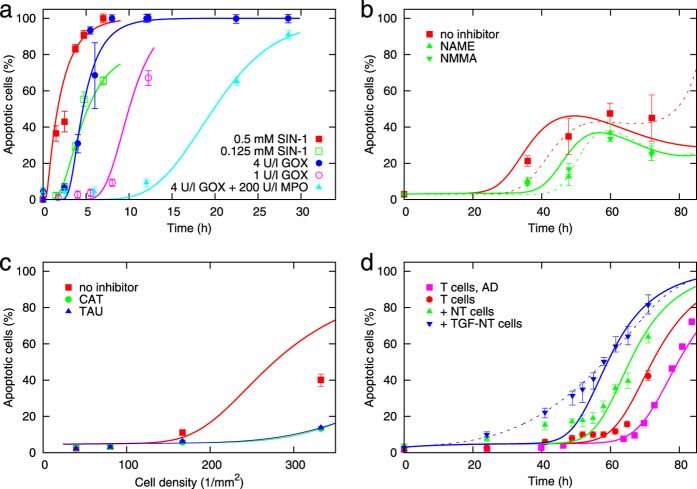
Model calculations representing data on apoptosis induction in 208F src3 transformed rat fibroblasts. Panel a: Apoptosis induced^24^ by externally added 0.5 or 0.125 mM of 13-morpholinosydnonimine (SIN-1, a donor of peroxynitrite), 4 or 1 mU/ml glucose oxidase (GOX, donor of hydrogen peroxide), or 4 mU/ml GOX + 200 mU/ml myeloperoxidase (MPO), a system generating hypochlorous acid. Background apoptosis has been neglected. Panel b: Apoptosis induced^25^ in transformed 208F src3 cells upon signalling by normal 208F cells (9.6 cm^2^ wells with 40000 transformed cells with inserts with 40000 TGF-β pre-treated normal cells) without or with inhibitors of NO^·^ synthesis N-omega-Nitro-L-arginine methylester hydrochloride (NAME, 1.2 mM) or N6-methyl-L-arginine (NMMA, 1.2 mM). Panel c: Autocrine destruction of transformed cells in dependence on their seeding density^26^, without inhibitors or with catalase (CAT, 20 U/ml) accelerating the decay of hydrogen peroxide or with taurine (TAU, 25 mM) removing hypochlorous acid. Panel d: Kinetics of apoptosis in transformed cells^26^ in autocrine destruction (AD) studies (magenta, cells seeded at 170 cells/mm^2^) or in co-culture system with normal cells seeded 24 h beforehand (red, transformed cells only, 100 cells/mm^2^; green, with non-transformed cells seeded at 100 cells/mm^2^; blue, with non-transformed cells pre-treated with TGF-β). Points represent the means and errorbars their standard errors from several repeats of the experiments^24^,^25^,^26^; lines depict model calculations.

**Figure 3:**
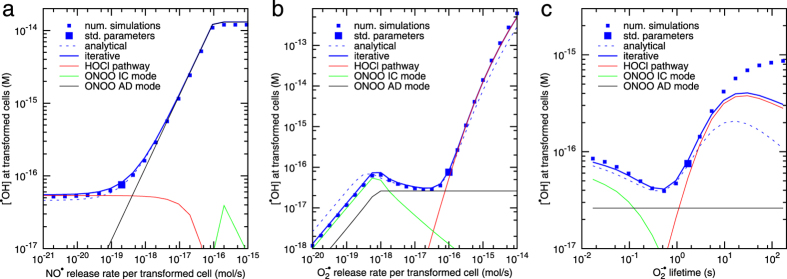
Predicted effectiveness of intercellular signalling leading to the induction of apoptosis in transformed cells in dependence on system parameters. The signalling effectiveness has been assessed in terms of ^·^OH concentrations at transformed cells. Shown are the results of detailed numerical simulations (squares) and their approximation by the iterative procedure (blue line) and by analytical formulas (dashed blue line). Separately indicated are also the contributions to ^·^OH yields from the HOCl pathway (red line) and from the peroxynitrite pathway in autocrine and inter-culture modes, i.e. with nitric oxide derived from transformed or normal cells (ONOO AD and IC modes, black and green lines, respectively). Around the standard values from Tables 2 and 3, depicted by the larger symbols, the following parameters have been varied: (**a**) the release rate of NO^·^ by transformed cells, (**b**) the release rate of O_2_^−·^ by transformed cells, and (**c**) the lifetime of O_2_^−·^. The effects of varying other model parameters are presented in Supplementary Material.

**Figure 4:**
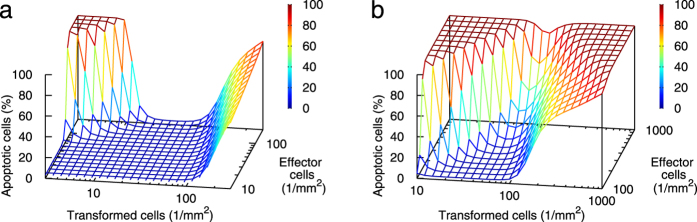
U-shaped (bell-shaped) curves are predicted for the percentage of apoptotic cells in dependence on the density of transformed cells at relatively high densities of normal cells. Simulated have been co-culture experiments with homogeneously seeded transformed cells in wells and TGF-β pre-treated normal cells in cell culture inserts, sharing common medium (3 mm height), at the indicated seeding densities, with inserts placed 1 mm above the wells. Results obtained with the standard parameters for normal and transformed rat fibroblasts (Tables 2 and 3) are shown for 24 h co-culture (panel a). Sharper U-shaped curves are obtained if higher cell densities are achievable (panel b, 24 h co-culture, parameter set A in Tables 2 and 3).

To help quantitatively understand the IIA phenomenon *in vitro* and its implications to carcinogenesis *in vivo*, a mechanistic model of the underlying intercellular signalling and the induction and execution of apoptosis in precancerous cells was developed^22^,^36^. The model explicitly accounts for apoptosis (either spontaneous or induced via the signalling) competing with cell proliferation, as well as for the removal of apoptotic bodies. Apoptosis induced upon IIA signalling is triggered by cellular damage (peroxidation of membrane lipids) which is initiated by attacks of inducers (^·^OH radicals). These are formed in a reaction cascade (Fig. 1 and Table 1) of intercellular signalling, starting from superoxide, nitric oxide, and peroxidase (primary signals). The biochemical reactions involved in this reaction cascade are accounted for by spatially inhomogeneous mass-action kinetics, with source terms for primary signals originating from signalling cells and sink terms for inducers of apoptosis which are removed upon attacking any of the cells. Reactions not explicitly included in the aforementioned reaction scheme such as the removal of ^·^OH radicals by proteins in the intercellular medium are represented by species’ lifetimes. Cells are capable of repairing the induced damage; a first-order repair process is assumed here. Intracellular transduction pathways leading to the execution of apoptosis, likely including positive and/or negative feedbacks, are not modelled explicitly but reflected by a non-linear, sigmoidal (ultrasensitive) response function of the induced damage. This paper is mainly focused on co-culture experiments, in which transformed cells are grown in wells and normal cells in inserts placed 1 mm above the wells, so that the two cultures share the growth medium but are physically separated^15^. For simplicity, a one-dimensional formalism has been used, describing cell populations not in terms of individual cells but cell densities and considering only the distance (*x*) from the transformed population instead of the full position vector. Throughout this work, constant per-cell release rates of primary signals are assumed; model extension to time-dependent releases is straightforward but at the price of introducing additional parameters. The cell densities can vary largely with time (*t*) due to proliferation and induction of apoptosis. Hence also the concentrations of signalling species are time-dependent. These concentrations are space-dependent too: finite lifetimes of the species limit their diffusion from the production sites, and mutual reactions further modify the resulting concentration profiles.

The intercellular signalling, induction of membrane damage, and triggering and execution of apoptosis are described by the following set of coupled differential equations^22^:


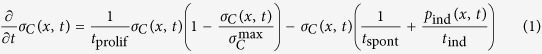



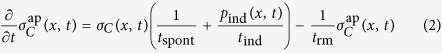



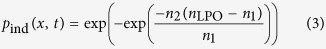



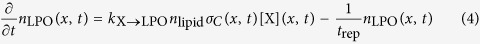



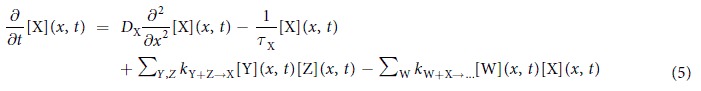



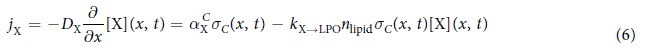


Equations (1, 2) describe the cell population dynamics, denoting the cell density by *σ*_*C*_; the position of transformed cells corresponds to *x* = 0 and that of normal cells to *x = L*_1_ (in typical co-culture experiments considered here, *L*_1_ = 1 mm). A density-inhibited cell proliferation has been accounted for by a logistic model, where *σ*_*C*_^max^ is the maximum cell density that corresponds to confluence; *t*_prolif_ is the characteristic time (i.e. inversed rate) of cell proliferation before this starts to be limited by the density inhibition. Both spontaneous and signalling-induced apoptosis are modelled, with characteristic times denoted by *t*_spont_ and *t*_ind_, respectively. A first-order removal of apoptotic cells (whose density is *σ*_*C*_^ap^) is assumed in equation (2), with characteristic time *t*_rm_. The probability of signalling-induced apoptosis (*p*_ind_) is described by a Gompertz sigmoidal function, equation (3), depending on the amount (*n*_LPO_) of peroxidative damage to membrane lipids; parameters *n*_1_ and *n*_2_ govern the position and slope of this sigmoidal function. Equation (4) describes how the amount of membrane damage increases with the attacks of inducers (^·^OH assumed here); the rate of damage induction is given by the local concentration, [X], of the inducer, by the amount (*n*_lipid_) of lipids per cell accessible to the damage, by cell density, and by the reaction rate constant (*k*_X→LPO_) for initiating lipid peroxidation by the inducer. A first-order repair process is considered, with rate 1/*t*_rep_. Equations (5, 6) describe the underlying biochemical signalling cascade: A set of reaction-diffusion Equations (5) describes the time- and space-dependent concentrations, [X](*x, t*), for inducers of apoptosis as well as all other species considered. Here, *D*_X_ stands for the species’ diffusion coefficient, *τ*_X_ its lifetime (related to the species’ half-life by *τ*^X^_1/2_ = *τ*_X_ln(2)), and *k*_Y+Z→X_ and *k*_W+X→…_ denote reaction rate constants in which species X is produced or consumed, respectively; the considered reactions are listed in Table 1. The corresponding boundary conditions for species’ fluxes are given by Equation (6), with *α*_X_^*C*^ denoting the per-cell release rate of the species.

### Multi-scale approach

The given signalling involves extremely short-lived species such as ^·^OH with lifetimes in the range of microseconds but also relatively stable species such as H_2_O_2_ or POD with lifetimes in the range of hours to days (Table 3). The execution of apoptosis up to the endpoint of morphological manifestation, even by very high levels of inducers, takes >1 h, and the cells double about once a day *in vitro* (Table 2). However, the involved temporal scales (microseconds, milliseconds, seconds, and hours to days) may be separated, since short-lived species reach, within the period of about 5–10 lifetimes, their quasi-steady-state concentration profiles that are given by interim cell densities and concentration profiles of longer-lived species (results of detailed test simulations not shown). In particular, simulations that assess the time- and space-dependent concentrations of the involved species might have been separated from the simulations of cellular population dynamics. This separation works even for the relatively stable H_2_O_2_, as peroxidase levels of the order of 10^−8^ M, found relevant for the given system^22^, reduce the estimated lifetime of H_2_O_2_ from 2.7 h to effectively about 4 s only (cf. Equation (9b) in Supplementary Material). Peroxidase, on the other hand, is a relatively stable enzyme produced slowly by the signalling cells, so that it only gradually accumulates in the system, and its concentration profiles have to be simulated explicitly within the cell dynamics step.

### Numerical simulations

The given set of coupled differential equations has been simulated numerically using the forward-time central-space finite difference method implemented in a dedicated FORTRAN code^22^ running on a high-end LINUX PC. The multi-scale approach has enabled using multiple time steps and spatial grid sizes in the simulations of species’ diffusion and mutual reactions, as well as in the induction and execution of apoptosis. The smallest grid size has been related to the diffusion length for the shortest-lived species (denoted by subscript A; typically ^·^OH) by ∆_A_*x* = (*D*_A_*τ*_A_)^1/2^/5, and the shortest time step has been taken as ∆_A_*t* = (∆_A_*x*)^2^/(2*D*_A_); this choice (including the safety factor of 5 in ∆_A_*x*) fulfils the stability criteria while keeping the simulation times reasonably low^22^. As lifetimes and to a smaller extent also diffusion coefficients vary among the species considered, enlarged grid sizes and prolonged time steps might have been used for longer-lived species. The time steps have been, however, reduced to correctly account for the consumption of the given species in mutual reactions, using analytical estimates of concentrations of its reaction partners that are discussed below. Similarly, time steps much shorter than the characteristic times for cell proliferation and execution of apoptosis have been taken when simulating these processes. Where only quasi-stationary steady-states (concentration profiles) are reported, only Equations (5, 6) have been solved; here, cell densities and the levels of peroxidase have been fixed at 100 cells/mm^2^ and 10^−8^ M, respectively, relevant for the given system^22^. Simulations on the kinetics of IIA have been performed by combining numerical simulations of cell population dynamics, Equations (1, 2, 3, 4), with iterative solutions for the signalling cascade, Equations (5, 6), which are described below.

### Analytical approximations to concentration profiles of signalling species

The results of the numerical simulations have been approximated by an analytical approach in order to enable quick on-the-fly calculations of the quasi-steady-state concentrations of signalling species, in particular apoptosis-inducing ^·^OH, for studies on the population dynamics. Furthermore, once benchmarked accordingly, analytical formulas enable interpolations between and to some extent also extrapolations from simulation results obtained for largely varying parameter values, needed to deal with the parameter identifiability issues and to cover the biologically relevant parameter space.

In these analytical approximations, reactions with relatively long-lived species have been accounted for as if these long-lived species were distributed homogeneously in space, i.e. via correspondingly reducing the effective lifetime τ* of the shorter-lived species, e.g. for H_2_O_2_ removed by POD as mentioned above. Mutual reactions between species with comparable lifetimes have been approximated by local absorption terms, e.g. for the dismutation of superoxide or for the reaction of superoxide with nitric oxide. Neglecting the competition for superoxide between individual signalling pathways, analytical formulas have been derived. Details on the method and the resulting formulas are given in Supplementary Material.

### Iterative approximations to concentration profiles

To more accurately account for the effects of mutual reactions and in particular for the interplay between the pathways and their competition for superoxide, an iterative approach based on the perturbation theory has been used. It starts from concentration profiles ([A]^(0)^, [B]^(0)^, …) of signalling species that would occur if there were no mutual reactions at all. In the first iterative step, refined concentration profiles ([A]^(1)^, [B]^(1)^, …) are derived, approximating the reactions by local absorption terms or by reduced effective lifetimes as described above; here e.g. [A]^(1)^ is calculated using zero-step concentrations ([B]^(0)^, …) of the reaction partners of species A. The first iteration thus corresponds to the above-described analytical approach. In an analogous way the procedure continues to higher-order terms,


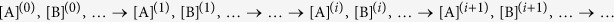


Details on the method are given in Supplementary Material.

## Additional Information

**How to cite this article**: Kundrát, P. and Friedland, W. Enhanced release of primary signals may render intercellular signalling ineffective due to spatial aspects. *Sci. Rep.* **6**, 33214; doi: 10.1038/srep33214 (2016).

## Supplementary Material

Supplementary Information

## Figures and Tables

**Table 1:** Reactions involved in IIA signalling taken into account in the present work.

No.	Reaction	Rate constant	Reference
1	O_2_^−·^ + NO^·^ → ONOO^−^	6 × 10^9^ M^−1^ s^−1^	37
2	O_2_^−·^ + O_2_^−·^ + 2 H^+^ → H_2_O_2_ + O_2_	2 × 10^5^ M^−1^ s^−1^	38
3	H_2_O_2_ + POD → POD-I	2.6 × 10^7^ M^−1^ s^−1^	39
4	H_2_O_2_ + POD-I → POD + H_2_O + O_2_	2 × 10^6^ M^−1^ s^−1^	39
5	Cl^−^ + POD-I → POD + HOCl	2.5 × 10^4^ M^−1^ s^−1^	39
6	HOCl + POD → POD-I + Cl^−^	2.4 × 10^7^ M^−1^ s^−1^	40
7	HOCl + O_2_^−·^ → ^·^OH + Cl^−^ + O_2_	7.5 × 10^6^ M^−1^ s^−1^	41
8	H_2_O_2_ + HOCl → H_2_O + O_2_ + H^+^ Cl^−^	1 × 10^5^ M^−1^ s^−1^	24
9	NO^·^ + NO^·^ (+O_2_) → 2 NO_2_^·^	8 × 10^3^ M^−1^ s^−1^	42, assuming [O_2_] = 1mM^43^
10	NO_2_^·^ + NO^·^ → N_2_O_3_	1.1 × 10^9^ M^−1^ s^−1^	44
11	N_2_O_3_ → NO_2_^·^ + NO^·^	8.4 × 10^4^ s^−1^	44
12	N_2_O_3_ + HOO^−^ → HNO_2_ + ONOO^−^	1 × 10^9^ M^−1^ s^−1^	45, taking pKa H_2_O_2_ = 11.7
13	^·^OH + lipid → initiation of lipid peroxidation	1 × 10^9^ M^−1^ s^−1^	46

**Table 2:** Values of basic cell biology parameters used in this work.

		Value			
Parameter		Standard	Set A	Set B	Set C
*σ*^max^_T_	Maximal density of transformed cells (cells/mm^2^)	300	1000	1000	
*σ*^max^_NT_	Maximal density of non-transformed cells (cells/mm^2^)	200	1000	1000	
*t*_prolif_	Characteristic time of cell proliferation (h)	22			
*t*_spont_	Characteristic time of spontaneous apoptosis (h)	225			
*t*_ind_	Characteristic time of apoptosis induced by the signalling (h)	1.7			
*n*_lipid_	Amount of lipids in cell membrane accessible to damage (mol/cell)	10^−15^			
*n*_1_	Characteristic level of membrane damage leading to induction of apoptosis (mol/cell)	10^−17^		7 × 10^−17^	
*n*_2_	Rate of change in the probability of apoptosis induction with membrane damage (1)	3.5			2.2
*t*_rep_	Characteristic time for the repair of membrane damage (h)	15	12	N.A.	
*t*_rm_	Characteristic time for the removal of apoptotic cells (h)	15	12		

Standard parameter values have been estimated by a simultaneous analysis of data^24^,^25^ on apoptosis induction in transformed rat fibroblasts exposed to defined donors of signalling species or challenged by co-culture with non-transformed cells^22^,^28^. In addition, modified parameter sets (Sets A, B and C) are reported that have been used in alternative analyses of individual data, as mentioned in the text; where no value is given, the standard one has been kept in the alternative set as well.

**Table 3:** Diffusion coefficients, lifetimes and per-cell release rates of IIA signalling species.

Species	Diffusion coefficient (dm^2^/s)	Lifetime *in vitro*	Per-cell release rate					
			Transformed cells (mol/s)				Normal cells, relative to transformed cells (1)	TGF-β pre-treated normal cells, relative to transformed cells (1)
			Standard	Set A	Set B	Set C		
O_2_^−·^	2.8 × 10^−7^	1.7 s	10^−16^	8 × 10^−17^	7 × 10^−17^	10^−19^	—	—
NO^·^	3.3 × 10^−7^	180 s	2 × 10^−19^			7 × 10^−20^	1	10
ONOO^−^	1.5 × 10^−7^	4.2 s	—				—	—
H_2_O_2_	2.3 × 10^−7^	2.7 h	—				—	—
POD	7.0 × 10^−9^	12 d	10^−22^			5 × 10^−20^	1	10
HOCl	1.0 × 10^−7^	38 ms	—				—	—
^·^OH	2.2 × 10^−7^	3.4 μs	—				—	—

Diffusion coefficients have been taken from the literature^22^,^28^. Lifetimes and standard release rates of the species are previous estimates^22^,^28^ from a simultaneous analysis of data on apoptosis induction in transformed rat fibroblasts exposed to externally added signals or challenged by co-culture with non-transformed cells^24^,^25^. In addition, modified release rates (Sets A, B and C) are reported that have been used in alternative analyses of individual data mentioned in the text; where no value is given, the standard one has been kept in the alternative set. In all simulations, normal cells have been assumed to release the same amounts of nitric oxide and peroxidase as transformed cells, but to produce no superoxide, and to enhance the release rates ten times upon pre-treatment with TGF-β.
