# Supplementary material for: Enhanced release of primary signals may render intercellular signalling ineffective due to spatial aspects
Source: Sci Rep. 2016 Sep 20;6:33214. doi: 10.1038/srep33214 (PMC5028836; doi:10.1038/srep33214)
Supplement: Supplementary Information [file srep33214-s1.pdf]

Supplementary Material

Enhanced release of primary signals may render intercellular signalling ineffective due to spatial aspects

Pavel Kundrát, Werner Friedland

Institute of Radiation Protection, Department of Radiation Sciences, Helmholtz Zentrum München GmbH - German Research Center for Environmental Health, Neuherberg, Germany

1. Methods

1.1 Multi-scale approach and quasi-steady-state approximations

The given biochemical signalling cascade (Fig.1 and Tab.1-3 in the main text) includes species with very different lifetimes. This makes numerical simulations of the corresponding set of reaction-diffusion equations expensive in terms of computation time: Very short time steps and small spatial grid sizes are needed for the short-lived species for the sake of accuracy, while the system has to be followed over long periods of time to account for long-lived species and cell proliferation and apoptosis.

However, even if starting from initial conditions of no signals present in the medium, short-lived species reach relatively quickly, within ≈5-10 times their lifetime, a quasi-steady-state concentration profile that is given by their production rates by cells or through reactions of longer-lived species. Concentrations of hydroxyl radicals, for instance, do not change over µs – ms time scale, and follow the interim levels of peroxynitrite, hypochlorous acid and superoxide that vary over longer time scales (Tab.3 in the main text). This observation enables using a multi-scale modelling approach that is for individual species described in detail below.

1.1.1 Hydroxyl radicals

Hydroxyl radicals ($^{\bullet}$OH) are extremely short-lived (highly reactive) species; therefore, their diffusion has been neglected here. From the reaction-diffusion equations (Eq.5 in the main text), their quasi-steady-state concentration profile can be derived, for which $\frac{\partial}{\partial t}[\,^{\bullet}\mathrm{OH}\,](x,T) \approx 0$ over timescales *T* much longer than τ_{OH} but short compared to the lifetimes of longer-lived species and to characteristic times of cellular processes such as proliferation or apoptosis:

$$[\,^{\bullet}\mathrm{OH}\,](x,T) \approx \left(\tau_{\mathrm{OH}} k_{\mathrm{HOCl+O_2^{-\bullet}}}[\mathrm{HOCl}](x,T)[\mathrm{O_2^{-\bullet}}](x,T) + \frac{\tau_{\mathrm{OH}}\eta_{\mathrm{OH}}}{\tau_{\mathrm{ONOO}}}[\mathrm{ONOO}](x,T)\right) f_{\mathrm{LPO}}(x,T). \quad (1)$$

Here the first term describes the production of hydroxyl radicals upon the reaction of hypochlorous acid with superoxide (peroxidase pathway). The second term captures $^{\bullet}$OH from peroxynitrite pathway, i.e. from the decay of ONOOH, the conjugate acid to peroxynitrite anion $\mathrm{ONOO^-}$, with lifetime τ_{ONOO} and efficiency η_{OH}~33 % [Lobachev and Rudakov 2006] of decaying into $^{\bullet}$OH and $\mathrm{NO_2^{\bullet}}$. The arguments (x,T) emphasize the (space-dependent, *x*) quasi-steady-state approximation, i.e. time-dependence over time scales *T* on which HOCl and peroxynitrite levels vary but time-independence over shorter time scales. The factor f_{LPO} accounts for the absorption of $^{\bullet}$OH upon initiation of lipid peroxidation (LPO) in cell membranes; it holds [Kundrát et al 2012]

$$f_{\mathrm{LPO}}(x,T) = \frac{1}{1+\frac{n_{\mathrm{lipid}}\tau_{\mathrm{OH}}}{\sqrt{D_{\mathrm{OH}}\tau_{\mathrm{OH}}}}k_{\mathrm{LPO}}\,\sigma(x,T)}\,, \tag{2}$$

where $k_{\mathrm{LPO}} = 10^9\ \mathrm{M}^{-1}\ \mathrm{s}^{-1}$ [Radi et al 1991] stands for the reaction rate constant for lipid peroxidation by $^{\bullet}$OH, $n_{\mathrm{lipid}} \approx 10^{-15}$ mol/cell [Alberts et al 2002) for the amount of lipid molecules in cell membrane that could be attacked, and $\sigma(x,T)$ for cell density at time T, at x = 0 for transformed cells and at $x = L_1$ ~ 1 mm for effector cells in co-culture experiments.

1.1.2 Hypochlorous acid

Hypochlorous acid is also relatively short-lived, quickly reacting with constituents of intercellular medium [Kundrát et al 2012, Deborde and von Gunten 2008]. Hence its diffusion can be neglected with respect to its lifetime, and for time scales T long compared to HOCl lifetime it holds

$$[\mathrm{HOCl}](x,T) \approx \tau^*_{\mathrm{HOCl}}k_{\mathrm{H_2O_2+POD}}[\mathrm{H_2O_2}](x,T)[\mathrm{POD}](x,T)\,; \tag{3}$$

here τ^*_{HOCl} is the lifetime of HOCl reduced to account for its absorption in reaction with superoxide,

$$\tau^*_{\mathrm{HOCl}} = \frac{1}{\left(\frac{1}{\tau^*_{\mathrm{HOCl}}}+k_{\mathrm{HOCl+O_2^{-\bullet}}}[\mathrm{O_2^{-\bullet}}](0,T)\right)}\,, \tag{3b}$$

where superoxide concentration at x=0 has been taken as an upper limit on superoxide levels (see next subsection), somewhat overestimating the effect of the reaction on HOCl lifetime. The absorption of HOCl in reaction with H_2O_2 and POD has been neglected. Assuming peroxidase be a relatively stable enzyme [Kundrát et al 2012], it is distributed almost homogeneously, $[\mathrm{POD}](x,T) \approx [\mathrm{POD}](T)$.

1.1.3 Superoxide

For steady-state concentration profile of superoxide, the reaction-diffusion equation (Eq.5 in the main text) takes the form of

$$0 \approx D_{\mathrm{O_2^{-\bullet}}}\frac{\partial^2}{\partial x^2}[\mathrm{O_2^{-\bullet}}](x,T) - \frac{1}{\tau_{\mathrm{O_2^{-\bullet}}}}[\mathrm{O_2^{-\bullet}}](x,T) - 2k_{dism}[\mathrm{O_2^{-\bullet}}]^2(x,T) - k_{\mathrm{O_2^{-\bullet}+NO^{\bullet}}}[\mathrm{O_2^{-\bullet}}](x,T)[\mathrm{NO^{\bullet}}](x,T) - k_{\mathrm{HOCl+O_2^{-\bullet}}}[\mathrm{HOCl}](x,T)[\mathrm{O_2^{-\bullet}}](x,T)\,. \tag{4}$$

The boundary conditions are

$$j_0 = -D_{\mathrm{O_2^{-\bullet}}}\frac{\partial}{\partial x}[\mathrm{O_2^{-\bullet}}](0,T) = \alpha^{TC}_{\mathrm{O_2^{-\bullet}}}\sigma_{TC}(T)\,, \qquad j_L = -D_{\mathrm{O_2^{-\bullet}}}\frac{\partial}{\partial x}[\mathrm{O_2^{-\bullet}}](L,T) = 0\,, \tag{5}$$

where $\alpha^{TC}_{\mathrm{O_2^{-\bullet}}}$ stands for the release rate of superoxide per transformed cell (assumed to be constant) and $\sigma_{TC}(T)$ for the density of transformed cells, which is time-dependent due to cell proliferation and apoptosis.

Neglecting the effect of reactions and assuming that the diffusion length of superoxide $r_{\mathrm{O_2^{-\bullet}}} = \sqrt{D_{\mathrm{O_2^{-\bullet}}}\tau_{\mathrm{O_2^{-\bullet}}}}$ is much smaller than the height of cell culture medium, $r_{\mathrm{O_2^{-\bullet}}} \ll L$ ~ 3 mm, the steady-state concentration profile of superoxide is

$$[\mathrm{O_2^{-\bullet}}](x,T) \approx \alpha^{TC}_{\mathrm{O_2^{-\bullet}}}\sigma_{TC}(T)\frac{\tau_{\mathrm{O_2^{-\bullet}}}}{r_{\mathrm{O_2^{-\bullet}}}}\mathrm{e}^{-x/r_{\mathrm{O_2^{-\bullet}}}}\,. \tag{6}$$

In particular, at *x*=0 where transformed cells are, $[O_2^{-\bullet}](0,T) \approx \alpha^{TC}_{O_2^{-\bullet}} \sigma_{TC}(T) \frac{\tau_{O_2^{-\bullet}}}{r_{O_2^{-\bullet}}}$.

1.1.4 Nitric oxide

Nitric oxide is produced by transformed cells (at *x*=0, cell density σ_{TC}, per-cell release rate $\alpha^{TC}_{NO^\bullet}$) as well as by effector cells (at $x=L_1$, cell density σ_E, per-cell release rate $\alpha^{EC}_{NO^\bullet}$), so that the boundary conditions read

$$j_0 = -D_{NO^\bullet} \frac{\partial}{\partial x}[NO^\bullet](0,T) = \alpha^{TC}_{NO^\bullet} \sigma_{TC}(T)\,, \qquad j_{L_1} = D_{NO^\bullet}\left(\frac{\partial}{\partial x_+} - \frac{\partial}{\partial x_-}\right)[NO^\bullet](L_1,T) = \alpha^{EC}_{NO^\bullet} \sigma_{EC}(T)\,,$$
$$j_L = -D_{NO^\bullet} \frac{\partial}{\partial x}[NO^\bullet](L,T) = 0\,, \tag{7}$$

where $\frac{\partial}{\partial x_+}$ and $\frac{\partial}{\partial x_-}$ denote right and left derivatives.

If the effect of reactions is neglected and the diffusion length $r_{NO^\bullet} = \sqrt{D_{NO^\bullet} \tau_{NO^\bullet}}$ is much smaller than the height of cell culture medium, $r_{NO^\bullet} \ll L$, we obtain

$$[NO^\bullet](x,T) \approx \alpha^{TC}_{NO^\bullet} \sigma_{TC}(T) \frac{\tau_{NO^\bullet}}{r_{NO^\bullet}} e^{-\frac{x}{r_{NO^\bullet}}} + \frac{1}{2} \alpha^{EC}_{NO^\bullet} \sigma_{EC}(T) \frac{\tau_{NO^\bullet}}{r_{NO^\bullet}} e^{-\frac{(L_1-x)}{r_{NO^\bullet}}}\,. \tag{8}$$

Note the factor of 1/2 in the second term, which accounts for the diffusion from the source at $x=L_1$ into two directions.

1.1.5 Hydrogen peroxide

For hydrogen peroxide the quasi-steady-state approximation to the reaction-diffusion equation reads

$$0 \approx D_{H_2O_2} \frac{\partial^2}{\partial x^2}[H_2O_2](x,T) - \frac{1}{\tau_{H_2O_2}}[H_2O_2](x,T) + k_{dism}[O_2^{-\bullet}]^2(x,T) - k_{H_2O_2+POD}[H_2O_2](x,T)[POD](x,T) -$$
$$k_{HOCl+H_2O_2}[HOCl](x,T)[H_2O_2](x,T)\,. \tag{9}$$

As discussed in Sec.1.1.2, $[POD](x,T) \approx [POD](T)$, so that the removal of H_2O_2 by peroxidase could be included in the effective lifetime of H_2O_2,

$$\tau^*_{H_2O_2} = \frac{1}{k_{H_2O_2+POD}[POD](T) + \frac{1}{\tau_{H_2O_2}}}\,. \tag{9b}$$

Neglecting other reactions in which H_2O_2 is removed, such as the reaction with HOCl listed in Eq.9, we have

$$0 \approx D_{H_2O_2} \frac{\partial^2}{\partial x^2}[H_2O_2](x,T) - \frac{1}{\tau^*_{H_2O_2}}[H_2O_2](x,T) + k_{dism}[O_2^{-\bullet}]^2(x,T)\,. \tag{10}$$

For the simple form of superoxide concentration profile given by Eq.6, this non-homogeneous differential equation can be solved analytically, as a general solution of the homogeneous equation (without the source term, i.e. the dismutation term) plus a particular solution reflecting the source term. Assuming that the effective diffusion distance of hydrogen peroxide, $r^*_{H_2O_2} = \sqrt{D_{H_2O_2} \tau^*_{H_2O_2}}$, be much smaller than the size of the region of interest *L* (which for sufficiently high levels of peroxidase is fulfilled), after a few algebraic manipulations we obtain for the levels of hydrogen peroxide at transformed cells

$$[\mathrm{H_2O_2}](0,T) \approx k_{dism}\,[\mathrm{O_2^{-\bullet}}]^2(0,T)\ \ \tau^*{}_{\mathrm{H_2O_2}}\ \frac{1}{1+2\frac{r^*{}_{\mathrm{H_2O_2}}}{r_{\mathrm{O_2^{-\bullet}}}}}\,. \qquad (11)$$

This result could be interpreted as if hydrogen peroxide were produced by dismutation of superoxide locally at *x*=0 only and accumulated over the period of $\tau^*{}_{\mathrm{H_2O_2}}$. Note however the non-trivial factor $1+2\frac{r^*{}_{\mathrm{H_2O_2}}}{r_{\mathrm{O_2^{-\bullet}}}}$ that accounts for the diffusion properties of superoxide and its dismutation product, hydrogen peroxide.

1.1.6 Antioxidants converting superoxide to hydrogen peroxide

The concept of superoxide lifetime used above accounts for reactions not explicitly included in the given reaction scheme that remove superoxide from the signalling. Numerous antioxidant systems, however, act via converting superoxide to hydrogen peroxide. This is the case e.g. for superoxide dismutase (SOD) or vitamin C [Gray and Carmichael 1992, Saran and Bors 1994].

With such antioxidants, e.g. SOD, the time- and space-dependent concetration of superoxide is given by

$$0 \approx D_{\mathrm{O_2^{-\bullet}}}\frac{\partial^2}{\partial x^2}[\mathrm{O_2^{-\bullet}}](x,T) - \frac{1}{\tau_{\mathrm{O_2^{-\bullet}}}}[\mathrm{O_2^{-\bullet}}](x,T) - 2k_{dism}[\mathrm{O_2^{-\bullet}}]^2(x,T) - k_{\mathrm{O_2^{-\bullet}+SOD}}[\mathrm{O_2^{-\bullet}}](x,T)[\mathrm{SOD}](x,T) - k_{\mathrm{O_2^{-\bullet}+NO^\bullet}}[\mathrm{O_2^{-\bullet}}](x,T)[\mathrm{NO^\bullet}](x,T) - k_{\mathrm{HOCl+O_2^{-\bullet}}}[\mathrm{HOCl}](x,T)[\mathrm{O_2^{-\bullet}}](x,T)\,, \qquad (4')$$

where $k_{\mathrm{O_2^{-\bullet}+SOD}}$ denotes the corresponding reaction rate constant, e.g. $6.4\times10^9\ \mathrm{M^{-1}\,s^{-1}}$ for Cu,Zn-SOD [Gray and Carmichael 1992]. For a spatially homogeneously distributed antioxidant, its removal of superoxide can be included in the effective lifetime of superoxide,

$$\tau^*_{\mathrm{O_2^{-\bullet}}} = \frac{1}{\frac{1}{\tau_{\mathrm{O_2^{-\bullet}}}} + k_{\mathrm{O_2^{-\bullet}+SOD}}[\mathrm{SOD}](T)}\ ,$$

and upon replacing $\tau_{\mathrm{O_2^{-\bullet}}}$ with $\tau^*_{\mathrm{O_2^{-\bullet}}}$ the above-discussed formulas for superoxide can be used.

For hydrogen peroxide, it holds

$$0 \approx D_{\mathrm{H_2O_2}}\frac{\partial^2}{\partial x^2}[\mathrm{H_2O_2}](x,T) - \frac{1}{\tau_{\mathrm{H_2O_2}}}[\mathrm{H_2O_2}](x,T) + k_{dism}[\mathrm{O_2^{-\bullet}}]^2(x,T) + k_{\mathrm{O_2^{-\bullet}+SOD}}[\mathrm{O_2^{-\bullet}}](x,T)[\mathrm{SOD}](T) - k_{\mathrm{H_2O_2+POD}}[\mathrm{H_2O_2}](x,T)[\mathrm{POD}](x,T) - k_{\mathrm{HOCl+H_2O_2}}[\mathrm{HOCl}](x,T)[\mathrm{H_2O_2}](x,T)\,. \qquad (9')$$

Analytical formulas can be derived in a way analogous to the one discussed in Sec.1.1.5, yielding

$$[\mathrm{H_2O_2}](0,T) \approx k_{dism}\,[\mathrm{O_2^{-\bullet}}]^2(0,T)\ \ \tau^*{}_{\mathrm{H_2O_2}}\ \frac{1}{1+2\frac{r^*{}_{\mathrm{H_2O_2}}}{r_{\mathrm{O_2^{-\bullet}}}}} + k_{\mathrm{O_2^{-\bullet}+SOD}}[\mathrm{SOD}](T)[\mathrm{O_2^{-\bullet}}](0,T)\,\tau^*{}_{\mathrm{H_2O_2}}\ \frac{1}{1+\frac{r^*{}_{\mathrm{H_2O_2}}}{r_{\mathrm{O_2^{-\bullet}}}}}\,. \qquad (11')$$

The first term describes the formation of hydrogen peroxide from superoxide by spontaneous dismutation; this process is quadratic in superoxide concentration, and hence leads to the factor of 2 in denominator. The second term describes dismutation catalysed by SOD (or other antioxidants); this conversion is linear in superoxide concentration, and hence the factor of 2 in denominator is not present.

1.2 Mutual reactions approximated by local absorption

In addition to having assumed that the diffusion length of superoxide be much smaller than the medium height, $r_{O_2^{-\bullet}} \ll L$, and hence having neglected factors such as $1-\mathrm{e}^{-L/r_{O_2^{-\bullet}}}$, the simple formula (6) for superoxide concentration neglects the effect of its reactions, namely its dismutation as well as reactions with $NO^\bullet$ and HOCl. These can however be accounted for in an analytical way by approximating the complex consumption patterns of mutual reactions by local absorption terms: The reaction of superoxide with nitric oxide is almost diffusion-limited, $k_{O_2^{-\bullet}+NO^\bullet}$=6×10^9 M^{-1} s^{-1} (Tab.1 in the main text). Superoxide is a short-lived species; its estimated lifetime *in vitro*, $\tau_{O_2^{-\bullet}}$=1.7 s (Tab.3), means a diffusion distance of $r_{O_2^{-\bullet}}$=69 µm only. Superoxide levels thus very quickly decrease with increasing distance from the transformed cells, and the dismutation term, $k_{dism}[O_2^{-\bullet}]^2$, is significant only in a close vicinity (of the order of $r_{O_2^{-\bullet}}$) of the transformed cells. Similarly, concentrations of nitric oxide decrease with increasing distance from its sources, i.e. transformed or effector cells. The reaction term $k_{O_2^{-\bullet}+NO^\bullet}[O_2^{-\bullet}](x,T)[NO^\bullet](x,T)$ in Eq.4 for superoxide (and in its analogue for nitric oxide) is thus non-negligible only in a close vicinity of transformed cells, for NO derived from transformed cells, or, for $NO^\bullet$ from effector cells, in the vicinity of the 'effective reaction point' L_r where $[O_2^{-\bullet}](L_r(T),T) = [NO^\bullet]_{EC}(L_r(T),T)$. More precisely, the effect of the dismutation term can be, using Eq.6 and the assumption $r_{O_2^{-\bullet}} \ll L$, evaluated as

$$\int_0^L k_{dism}[O_2^{-\bullet}]^2(x,T)\,\mathrm{d}x \approx \int_0^L k_{dism}\left(\alpha_{O_2^{-\bullet}}^{TC}\sigma_{TC}(T)\frac{\tau_{O_2^{-\bullet}}}{r_{O_2^{-\bullet}}}\mathrm{e}^{-\frac{x}{r_{O_2^{-\bullet}}}}\right)^2\mathrm{d}x$$

$$= k_{dism}\left(\alpha_{O_2^{-\bullet}}^{TC}\sigma_{TC}(T)\frac{\tau_{O_2^{-\bullet}}}{r_{O_2^{-\bullet}}}\right)^2 r_{O_2^{-\bullet}}\left(1-\mathrm{e}^{-\frac{L}{r_{O_2^{-\bullet}}}}\right) \approx k_{dism}[O_2^{-\bullet}]^2(0,T)r_{O_2^{-\bullet}}\,, \qquad (12)$$

i.e. as a product of the reaction rate constant, the concentrations of the reactants, and the effective size of the reaction region, which equals $r_{O_2^{-\bullet}}$ for superoxide dismutation.

Apart from $NO^\bullet$ autoxidation, the equations for $NO^\bullet$ are linear, so that $NO^\bullet$ derived from transformed and from effector cells could be treated separately. Similarly to the above-discussed case of superoxide dismutation, for the terms describing the reaction of superoxide with nitric oxide derived from transformed and effector cells, respectively, it holds (with |·| denoting absolute value of the argument):

$$\int_0^L k_{O_2^{-\bullet}+NO^\bullet}[O_2^{-\bullet}](x,T)[NO^\bullet]_{TC}(x,T)\,\mathrm{d}x \approx k_{O_2^{-\bullet}+NO^\bullet}[O_2^{-\bullet}](0,T)[NO^\bullet]_{TC}(0,T)\frac{2\,r_{O_2^{-\bullet}}\,r_{NO^\bullet}}{r_{O_2^{-\bullet}}+r_{NO^\bullet}}\,, \qquad (13)$$

$$\int_0^L k_{O_2^{-\bullet}+NO^\bullet}[O_2^{-\bullet}](x,T)[NO^\bullet]_{EC}(x,T)\,\mathrm{d}x \approx k_{O_2^{-\bullet}+NO^\bullet}[O_2^{-\bullet}](L_r,T)[NO^\bullet]_{EC}(0,T)\frac{2\,r_{O_2^{-\bullet}}\,r_{NO^\bullet}}{\left|r_{NO^\bullet}-r_{O_2^{-\bullet}}\right|}\,; \qquad (14)$$

the distinct signs in denominators follow from the exponentials in the two contributions to the concentration profile for nitric oxide, Eq.8.

Thus, instead of including these reaction terms in the reaction-diffusion equations directly, they could be approximately accounted for via boundary conditions. In this local absorption approximation, the quasi-steady-state concentration profiles of superoxide and nitric oxide are thus given as solutions to the following set of differential equations and boundary conditions:

$$0 = \frac{\partial^2}{\partial x^2}[O_2^{-\bullet}](x,T) - \frac{1}{r^2_{O_2^{-\bullet}}}[O_2^{-\bullet}](x,T) \qquad (15a)$$

$$\frac{\partial}{\partial x}[\mathrm{O}_2^{-\bullet}](0,T) = -\frac{\alpha_{\mathrm{O}_2^{-\bullet}}^{TC}\sigma_{TC}(T)}{D_{\mathrm{O}_2^{-\bullet}}} + \frac{\beta_0}{r_{\mathrm{O}_2^{-\bullet}}}[\mathrm{O}_2^{-\bullet}](0,T) \tag{15b}$$

$$\left(\frac{\partial}{\partial x_+} - \frac{\partial}{\partial x_-}\right)[\mathrm{O}_2^{-\bullet}](L_r,T) = +\frac{\beta_r}{r_{\mathrm{O}_2^{-\bullet}}}[\mathrm{O}_2^{-\bullet}](L_r,T) \tag{15c}$$

$$\frac{\partial}{\partial x}[\mathrm{O}_2^{-\bullet}](L,T) = 0 \tag{15d}$$

$$\beta_0 = \frac{k_{\mathrm{O}_2^{-\bullet}+\mathrm{NO}^\bullet}}{D_{\mathrm{O}_2^{-\bullet}}}\frac{2\,r_{\mathrm{O}_2^{-\bullet}}\,r_{\mathrm{NO}^\bullet}}{r_{\mathrm{O}_2^{-\bullet}}+r_{\mathrm{NO}^\bullet}}[\mathrm{NO}^\bullet]_{TC}(0,T) + \frac{2k_{dism}}{D_{\mathrm{O}_2^{-\bullet}}}\,r_{\mathrm{O}_2^{-\bullet}}\,[\mathrm{O}_2^{-\bullet}](0,T) \tag{15e}$$

$$\beta_r = \frac{k_{\mathrm{O}_2^{-\bullet}+\mathrm{NO}^\bullet}}{D_{\mathrm{O}_2^{-\bullet}}}\frac{2\,r_{\mathrm{O}_2^{-\bullet}}\,r_{\mathrm{NO}^\bullet}}{\left|r_{\mathrm{NO}^\bullet}-r_{\mathrm{O}_2^{-\bullet}}\right|}[\mathrm{NO}^\bullet]_{EC}(L_r,T) \tag{15f}$$

$$0 = \frac{\partial^2}{\partial x^2}[\mathrm{NO}^\bullet]_{TC}(x,T) - \frac{1}{r^2{}_{\mathrm{NO}^\bullet}}[\mathrm{NO}^\bullet]_{TC}(x,T) \tag{16a}$$

$$\frac{\partial}{\partial x}[\mathrm{NO}^\bullet]_{TC}(0,T) = -\frac{\alpha_{\mathrm{NO}^\bullet}^{TC}\sigma_{TC}(T)}{D_{\mathrm{NO}^\bullet}} + \frac{\gamma_0}{r_{\mathrm{NO}^\bullet}}[\mathrm{NO}^\bullet]_{TC}(0,T) \tag{16b}$$

$$\frac{\partial}{\partial x}[\mathrm{NO}^\bullet]_{TC}(L,T) = 0 \tag{16c}$$

$$\gamma_0 = \frac{k_{\mathrm{O}_2^{-\bullet}+\mathrm{NO}^\bullet}}{D_{\mathrm{NO}^\bullet}}\frac{2\,r_{\mathrm{O}_2^{-\bullet}}\,r_{\mathrm{NO}^\bullet}}{r_{\mathrm{O}_2^{-\bullet}}+r_{\mathrm{NO}^\bullet}}[\mathrm{O}_2^{-\bullet}](0,T) \tag{16d}$$

$$0 = \frac{\partial^2}{\partial x^2}[\mathrm{NO}^\bullet]_{EC}(x,T) - \frac{1}{r^2{}_{\mathrm{NO}^\bullet}}[\mathrm{NO}^\bullet]_{EC}(x,T) \tag{17a}$$

$$\frac{\partial}{\partial x}[\mathrm{NO}^\bullet]_{EC}(0,T) = 0 \tag{17b}$$

$$\left(\frac{\partial}{\partial x_+} - \frac{\partial}{\partial x_-}\right)[\mathrm{NO}^\bullet]_{EC}(L_r,T) = +\frac{\gamma_r}{r_{\mathrm{NO}^\bullet}}[\mathrm{NO}^\bullet]_{EC}(L_r,T) \tag{17c}$$

$$\left(\frac{\partial}{\partial x_+} - \frac{\partial}{\partial x_-}\right)[\mathrm{NO}^\bullet]_{EC}(L_1,T) = -\frac{\alpha_{\mathrm{NO}^\bullet}^{EC}\sigma_{EC}(T)}{D_{\mathrm{NO}^\bullet}} \tag{17d}$$

$$\frac{\partial}{\partial x}[\mathrm{NO}^\bullet]_{EC}(L,T) = 0 \tag{17e}$$

$$\gamma_r = \frac{k_{\mathrm{O}_2^{-\bullet}+\mathrm{NO}^\bullet}}{D_{\mathrm{NO}^\bullet}}\frac{2\,r_{\mathrm{O}_2^{-\bullet}}\,r_{\mathrm{NO}^\bullet}}{\left|r_{\mathrm{NO}^\bullet}-r_{\mathrm{O}_2^{-\bullet}}\right|}[\mathrm{O}_2^{-\bullet}](L_r,T) \tag{17f}$$

$$[\mathrm{NO}^\bullet]_{EC}(L_r(T),T) = [\mathrm{O}_2^{-\bullet}](L_r(T),T) \tag{18}$$

Equations 15-18 represent a set of coupled equations: Superoxide concentrations at x=0 and x=L_r obtained from Eq.15 affect the absorption terms γ_0 and γ_r in boundary conditions of Eqs. 16-17 for nitric oxide, and vice versa, nitric oxide levels influence superoxide concentrations through the absorption terms β_0 and β_r. The two species are also linked by Eq.18 that defines the effective reaction point L_r.

Two methods of solving this set of equations are described below. The first method is a relatively crude approximation, neglecting the competition for superoxide between the three signalling modes (peroxidase pathway and the peroxynitrite pathway with $NO^\bullet$ derived from transformed and effector cells, respectively).

However, it provides analytical formulas for quasi-steady-state concentration profiles of the signalling species and for the efficiencies of the distinct signalling modes relevant for intercellular induction of apoptosis. The second method accounts for the interplay of the pathways and modes via an iterative procedure.

1.3 Analytical formulas for quasi-steady-state concentration profiles and efficiencies of signalling modes

For constant absorption terms β_0, β_r, γ_0 and γ_r, analytical solutions can be found to Eqs.15a-d, 16a-c and 17a-e. The full formulas, keeping factors such as $1-\mathrm{e}^{-L/r_{\mathrm{O_2^{-\bullet}}}} \approx 1$, have been derived with Wolfram Mathematica® Version 9; however, here only their simplified versions are reported that neglect such factors, i.e. assume $r_{\mathrm{O_2^{-\bullet}}}, r_{\mathrm{NO^\bullet}} \ll L,\ L_1,\ L_r,\ L-L_1,\ L-L_r$:

$$[\mathrm{O_2^{-\bullet}}](0,T) \approx \frac{1}{1+\beta_0}\ \frac{\alpha^{TC}_{\mathrm{O_2^{-\bullet}}}\sigma_{TC}(T)\tau_{\mathrm{O_2^{-\bullet}}}}{r_{\mathrm{O_2^{-\bullet}}}} \tag{19a}$$

$$[\mathrm{O_2^{-\bullet}}](L_r,T) \approx \frac{1}{(1+\beta_r/2)(1+\beta_0)}\ \frac{\alpha^{TC}_{\mathrm{O_2^{-\bullet}}}\sigma_{TC}(T)\tau_{\mathrm{O_2^{-\bullet}}}}{r_{\mathrm{O_2^{-\bullet}}}}\ \mathrm{e}^{-L_r/r_{\mathrm{O_2^{-\bullet}}}} \tag{19b}$$

$$[\mathrm{NO^\bullet}]_{TC}(0,T) \approx \frac{1}{1+\gamma_0}\frac{\alpha^{TC}_{\mathrm{NO^\bullet}}\sigma_{TC}(T)}{r_{\mathrm{NO^\bullet}}}\tau_{\mathrm{NO^\bullet}} \tag{19c}$$

$$[\mathrm{NO^\bullet}]_{EC}(L_r,T) \approx \frac{1}{1+\gamma_r/2}\frac{\alpha^{EC}_{\mathrm{NO^\bullet}}\sigma_{EC}(T)}{2\,r_{\mathrm{NO^\bullet}}}\tau_{\mathrm{NO^\bullet}}\mathrm{e}^{-(L_1-L_r)/r_{\mathrm{NO^\bullet}}} \tag{19d}$$

$$L_r(T):\quad [\mathrm{NO^\bullet}]_{EC}(L_r,T) = [\mathrm{O_2^{-\bullet}}](L_r,T) \tag{19e=18}$$

Comparing these formulas with Eqs.6 and 8 reveals that the absorption terms at x=0 and x=L_r act as if they reduced the release rates of superoxide by (1+β_0) and (1+β_r/2), respectively; similarly for nitric oxide.

1.3.1 Hypochlorous acid pathway

For the HOCl pathway, neglecting in Eq.15 the reactions with $\mathrm{NO^\bullet}$, i.e. the terms containing $[\mathrm{NO^\bullet}]_{TC}(0,T)$ and $[\mathrm{NO^\bullet}]_{EC}(L_r,T)$, the absorption term β_0 contains only the dismutation term,

$$\beta_0 = \frac{2k_{dism}}{D_{\mathrm{O_2^{-\bullet}}}}\ r_{\mathrm{O_2^{-\bullet}}}\ [\mathrm{O_2^{-\bullet}}](0,T)\ . \tag{15e'}$$

Putting this into Eq.19a, we obtain a quadratic equation for $[\mathrm{O_2^{-\bullet}}](0,T)$, with positive solution

$$[\mathrm{O_2^{-\bullet}}](0,T) = \frac{\sqrt{1+8k_{dism}\alpha^{TC}_{\mathrm{O_2^{-\bullet}}}\ \sigma_{TC}(T)\frac{\tau^2{}_{\mathrm{O_2^{-\bullet}}}}{r_{\mathrm{O_2^{-\bullet}}}}}-1}{4k_{dism}\tau_{\mathrm{O_2^{-\bullet}}}}\ . \tag{20}$$

Using the approximation $\sqrt{1+x}-1 \approx \min(\sqrt{x},\ \frac{x}{2})$, illustrated in Fig.S1 (with min($\cdot$) standing for the minimum of the arguments), we may also write

$$[\mathrm{O_2^{-\bullet}}](0,T) \approx \min\left(\sqrt{\alpha^{TC}_{\mathrm{O_2^{-\bullet}}}\ \sigma_{TC}(T)/(2k_{dism}r_{\mathrm{O_2^{-\bullet}}})}\ ,\ \alpha^{TC}_{\mathrm{O_2^{-\bullet}}}\ \sigma_{TC}(T)\ \tau^2{}_{\mathrm{O_2^{-\bullet}}}/r_{\mathrm{O_2^{-\bullet}}}\right)\ . \tag{20'}$$

Combining Eqs.1, 2, 3, 9b, 11 and 20, we obtain the following analytical formulas for the concentration of hypochlorous acid:

$$[\mathrm{HOCl}](0,T) \approx \tau_{\mathrm{HOCl}} k_{dism} \left(\frac{\sqrt{1+8k_{dism}\alpha^{TC}_{\mathrm{O}_2^{-\bullet}}\,\sigma_{TC}(T)\frac{\tau^2_{\mathrm{O}_2^{-\bullet}}}{r_{\mathrm{O}_2^{-\bullet}}}}-1}{4k_{dism}\tau_{\mathrm{O}_2^{-\bullet}}} \right)^2 f_{\mathrm{POD}}\, \frac{1}{1+2\frac{r^*_{\mathrm{H_2O_2}}}{r_{\mathrm{O}_2^{-\bullet}}}}\ , \qquad (21a)$$

and for the efficiency of the HOCl pathway in terms of the levels of hydroxyl radicals attacking transformed cells:

$$[\ {}^{\bullet}\mathrm{OH}\ ]_{\mathrm{HOCl}}(0,T) \approx$$

$$\tau_{\mathrm{OH}}\tau_{\mathrm{HOCl}} k_{dism} k_{\mathrm{HOCl+O}_2^{-\bullet}} \left(\frac{\sqrt{1+8k_{dism}\alpha^{TC}_{\mathrm{O}_2^{-\bullet}}\,\sigma_{TC}(T)\frac{\tau^2_{\mathrm{O}_2^{-\bullet}}}{r_{\mathrm{O}_2^{-\bullet}}}}-1}{4k_{dism}\tau_{\mathrm{O}_2^{-\bullet}}} \right)^3 f_{\mathrm{POD}}\, \frac{1}{1+2\frac{r^*_{\mathrm{H_2O_2}}}{r_{\mathrm{O}_2^{-\bullet}}}}\, \frac{1}{1+\frac{n_{\mathrm{lipid}}\tau_{\mathrm{OH}}}{\sqrt{D_{\mathrm{OH}}\tau_{\mathrm{OH}}}} k_{\mathrm{LPO}}\,\sigma_{TC}(T)} \qquad (21b)$$

with

$$f_{\mathrm{POD}} = \frac{k_{\mathrm{H_2O_2+POD}}[\mathrm{POD}](T)}{k_{\mathrm{H_2O_2+POD}}[\mathrm{POD}](T)+1/\tau_{\mathrm{H_2O_2}}} \approx 1\ , \qquad (21c)$$

which for sufficiently large levels of POD and/or long lifetime of H_2O_2 (i.e. for $k_{\mathrm{H_2O_2+POD}}[\mathrm{POD}](T) \gg 1/\tau_{\mathrm{H_2O_2}}$) is approximately unity.

Note that with increasing density of transformed cells (or release rate of $O_2^{-\bullet}$), the amount of superoxide present in close vicinity of transformed cells increases (linearly at small and as a squared root at high transformed cell densities, Eq.20'), and hence the efficiency of the HOCl pathway rapidly increases, with up to the third power of transformed cell densities (or release rate of $O_2^{-\bullet}$, respectively). In agreement with this modelling result, the HOCl pathway has been found effective only when transformed cells were seeded at sufficiently high densities [Engelmann et al 2000, Portess 2007, Bechtel and Bauer 2009].

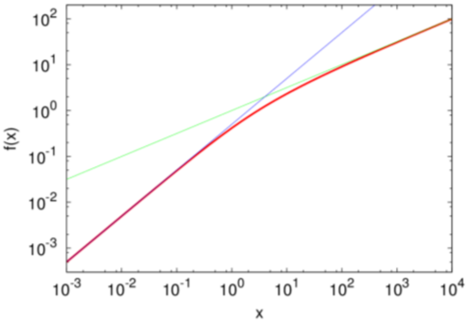

Figure S1: The function f(x) = $\sqrt{1+x}-1$ (red line) can be approximated by x/2 (blue line) for small x, say x < 1, and by $\sqrt{x}$ (green line) for large x, say x > 100. Taking the minimum of these two asymptotes serves as a reasonable approximation even in the intermediate region, overestimating the actual f(x) by less than a factor of two.

1.3.2 Peroxynitrite pathway

For the ONOO pathway, the approximation of the reaction between superoxide and nitric oxide by local absorption, at x=0 or at $x=L_r$ for nitric oxide derived from transformed and effector cells, respectively, also means that peroxynitrite is modelled as if produced locally at these points. The corresponding fluxes of peroxynitrite at x=0 or $x=L_r$ read

$$j_0(T) = k_{O_2^{-\bullet}+NO^\bullet}\ [O_2^{-\bullet}](0,T)\ [NO^\bullet]_{TC}(0,T)\, r_{0r}\ , \tag{22a}$$

$$j_r(T) = k_{O_2^{-\bullet}+NO^\bullet}\ [O_2^{-\bullet}](L_r,T)\ [NO^\bullet]_{EC}(L_r,T)\ r_{rr}\ , \tag{22b}$$

with the effective sizes of reaction region derived in Eqs.13-14, $r_{rr} = \frac{2\, r_{O_2^{-\bullet}}\, r_{NO^\bullet}}{\left| r_{NO^\bullet} - r_{O_2^{-\bullet}} \right|}$, $r_{0r} = \frac{2\, r_{O_2^{-\bullet}}\, r_{NO^\bullet}}{r_{NO^\bullet} + r_{O_2^{-\bullet}}}$.

Solving the diffusion equation for [ONOO] diffusing from x=L_r to x=0, we obtain

$$[ONOO]_{EC}(0,T) = j_r(T)\frac{\tau_{ONOO}}{r_{ONOO}}\ \frac{1+e^{-\frac{2L}{r_{ONOO}}}}{1-e^{-\frac{2L}{r_{ONOO}}}}\ e^{-\frac{L_r}{r_{ONOO}}} \approx j_r(T)\frac{\tau_{ONOO}}{r_{ONOO}}\ e^{-L_r/r_{ONOO}}\ , \tag{23a}$$

where we have assumed, similarly to the cases of superoxide and nitric oxide, that the diffusion length of peroxynitrite is negligible with respect to the medium height, $L \gg r_{ONOO}$.

Similarly, for the contribution to the peroxynitrite pathway from nitric oxide released by transformed cells,

$$[ONOO]_{TC}(0,T) \approx j_0(T)\ \frac{\tau_{ONOO}}{r_{ONOO}}. \tag{23b}$$

To be able to evaluate peroxynitrite levels in Eqs.23a-b, we thus need only the concentrations of superoxide and nitric oxide, at x=0 or at x=L_r for nitric oxide derived from transformed and effector cells, respectively. These are given by Eqs. 19a-19e. Unfortunately, even if the consumption of superoxide by its dismutation were neglected, Eqs. 19a-19e could not be solved analytically in their full form. Yet, if each pathway is considered separately, i.e. if their interplay through competing for superoxide is neglected, analytical results could be derived:

1.3.2.1 Autocrine mode of peroxynitrite pathway

For the autocrine mode of the ONOO pathway, i.e. both superoxide and nitric oxide derived from transformed cells, neglecting the interplay between the pathways/modes means putting β_r=γ_r=0 and neglecting the dismutation term in β_0 (Eq.15e). Combining Eqs.19a, 15e and 19c, we obtain a quadratic equation for superoxide levels at the transformed cells, with a unique positive solution

$$[O_2^{-\bullet}](0,T) = \frac{-1+\sqrt{1+\frac{4k_{O_2^{-\bullet}+NO^\bullet} r_{0r}\ \alpha^{TC}_{O_2^{-\bullet}}\ \sigma_{TC}(T)\frac{\tau_{O_2^{-\bullet}}}{r_{O_2^{-\bullet}}}\frac{\tau_{NO^\bullet}}{r_{NO^\bullet}}}{\left(1+k_{O_2^{-\bullet}+NO^\bullet} r_{0r}\left(\alpha^{TC}_{NO^\bullet}-\alpha^{TC}_{O_2^{-\bullet}}\right)\sigma_{TC}(T)\frac{\tau_{O_2^{-\bullet}}}{r_{O_2^{-\bullet}}}\frac{\tau_{NO^\bullet}}{r_{NO^\bullet}}\right)^2}}}{\frac{2k_{O_2^{-\bullet}+NO^\bullet}\ r_{0r}\ \frac{\tau_{NO^\bullet}}{r_{NO^\bullet}}}{\left|1+k_{O_2^{-\bullet}+NO^\bullet}\left(\alpha^{TC}_{NO^\bullet}-\alpha^{TC}_{O_2^{-\bullet}}\right)\sigma_{TC}(T) r_{0r}\frac{\tau_{O_2^{-\bullet}}}{r_{O_2^{-\bullet}}}\frac{\tau_{NO^\bullet}}{r_{NO^\bullet}}\right|}}\ . \tag{24a}$$

Similarly, from Eqs.19c, 16d and 19a, the concentration of nitric oxide reads

$$[NO^\bullet](0,T) = \frac{-1+\sqrt{1+\frac{4k_{O_2^{-\bullet}+NO^\bullet} r_{0r}\ \alpha^{TC}_{NO^\bullet}\ \sigma_{TC}(T)\frac{\tau_{O_2^{-\bullet}}}{r_{O_2^{-\bullet}}}\frac{\tau_{NO^\bullet}}{r_{NO^\bullet}}}{\left(1+k_{O_2^{-\bullet}+NO^\bullet} r_{0r}\left(-\alpha^{TC}_{NO^\bullet}+\alpha^{TC}_{O_2^{-\bullet}}\right)\sigma_{TC}(T)\frac{\tau_{O_2^{-\bullet}}}{r_{O_2^{-\bullet}}}\frac{\tau_{NO^\bullet}}{r_{NO^\bullet}}\right)^2}}}{\frac{2k_{O_2^{-\bullet}+NO^\bullet}\ r_{0r}\ \frac{\tau_{O_2^{-\bullet}}}{r_{O_2^{-\bullet}}}}{\left|1+k_{O_2^{-\bullet}+NO^\bullet} r_{0r}\left(-\alpha^{TC}_{NO^\bullet}+\alpha^{TC}_{O_2^{-\bullet}}\right)\sigma_{TC}(T)\frac{\tau_{O_2^{-\bullet}}}{r_{O_2^{-\bullet}}}\frac{\tau_{NO^\bullet}}{r_{NO^\bullet}}\right|}}\ . \tag{24b}$$

The concentration of peroxynitrite formed is given then by combining Eqs.24a-b with Eqs.22a and 23b. Applying $\sqrt{1+x}-1 \approx \min(\sqrt{x},\ \frac{x}{2})$, one can show that

$$[\mathrm{ONOO}]_{TC}(0,T) \approx \min\left(\alpha^{TC}_{\mathrm{NO}^\bullet}, \alpha^{TC}_{\mathrm{O}_2^-{}^\bullet}\right) \sigma_{TC}(T)\ \frac{\tau_{\mathrm{ONOO}}}{r_{\mathrm{ONOO}}}\ ; \tag{24c}$$

this approximation works in particular due to the large value of the reaction rate constant $k_{\mathrm{O}_2^-{}^\bullet+\mathrm{NO}^\bullet}$, as superoxide and nitric oxide react virtually whenever they encounter each other (almost diffusion-limited reaction). The amount of peroxynitrite formed is thus given directly by the production rates of superoxide or nitric oxide by transformed cells, whichever is smaller.

1.3.2.2 Inter-culture mode of peroxynitrite pathway

Neglecting β_0 in Eq.19b and requiring the amounts of superoxide and nitric oxide be equal at *x*=L_r (which is the definition of L_r, Eq.19e), Eq.19b yields a quadratic equation for $[\mathrm{O}_2^-{}^\bullet](L_r,T)$, with a single positive solution,

$$[\mathrm{O}_2^-{}^\bullet](L_r,T) = \frac{-1+\sqrt{1+2k_{\mathrm{O}_2^-{}^\bullet+\mathrm{NO}^\bullet}\ r_{rr}\ \alpha^{TC}_{\mathrm{O}_2^-{}^\bullet}\ \sigma_{TC}(T)\left(\frac{\tau_{\mathrm{O}_2^-{}^\bullet}}{r_{\mathrm{O}_2^-{}^\bullet}}\right)^2 \mathrm{e}^{-\frac{L_r}{r_{\mathrm{O}_2^-{}^\bullet}}}}}{k_{\mathrm{O}_2^-{}^\bullet+\mathrm{NO}^\bullet}\ r_{rr}\ \frac{\tau_{\mathrm{O}_2^-{}^\bullet}}{r_{\mathrm{O}_2^-{}^\bullet}}}\ . \tag{25}$$

Similarly, for the concentration of nitric oxide released from effector cells that is present at *x*=L_r one can derive

$$[\mathrm{NO}^\bullet]_{EC}(L_r,T) = \frac{-1+\sqrt{1+k_{\mathrm{O}_2^-{}^\bullet+\mathrm{NO}^\bullet}\ r_{rr}\ \alpha^{EC}_{\mathrm{NO}^\bullet}\ \sigma_{EC}(T)\left(\frac{\tau_{\mathrm{NO}^\bullet}}{r_{\mathrm{NO}^\bullet}}\right)^2 \mathrm{e}^{-\frac{L_1-L_r}{r_{\mathrm{NO}^\bullet}}}}}{k_{\mathrm{O}_2^-{}^\bullet+\mathrm{NO}^\bullet}\ r_{rr}\ \frac{\tau_{\mathrm{NO}^\bullet}}{r_{\mathrm{NO}^\bullet}}}\ . \tag{26}$$

Assuming that the reaction terms in Eqs.25-26 dominate, i.e. assuming

$$1 \ll 2k_{\mathrm{O}_2^-{}^\bullet+\mathrm{NO}^\bullet}\ r_{rr}\ \alpha^{TC}_{\mathrm{O}_2^-{}^\bullet}\ \sigma_{TC}(T)\left(\frac{\tau_{\mathrm{O}_2^-{}^\bullet}}{r_{\mathrm{O}_2^-{}^\bullet}}\right)^2 \mathrm{e}^{-\frac{L_r}{r_{\mathrm{O}_2^-{}^\bullet}}}$$

and

$$1 \ll k_{\mathrm{O}_2^-{}^\bullet+\mathrm{NO}^\bullet}\ r_{rr}\ \frac{\tau_{\mathrm{NO}^\bullet}}{r_{\mathrm{NO}^\bullet}}\ \alpha^{EC}_{\mathrm{NO}^\bullet}\ \sigma_{EC}(T)\left(\frac{\tau_{\mathrm{NO}^\bullet}}{r_{\mathrm{NO}^\bullet}}\right)^2 \mathrm{e}^{-\frac{L_1-L_r}{r_{\mathrm{NO}^\bullet}}}\ ,$$

so that $\sqrt{1+x}-1 \approx \sqrt{x}$ in Eqs.25-26, the point L_r at which superoxide and nitric oxide concentrations equal each other is approximated by

$$L_r(T) \approx \frac{r_{\mathrm{O}_2^-{}^\bullet}}{r_{\mathrm{O}_2^-{}^\bullet}+r_{\mathrm{NO}^\bullet}} L_1 + \frac{r_{\mathrm{O}_2^-{}^\bullet}\ r_{\mathrm{NO}^\bullet}}{r_{\mathrm{O}_2^-{}^\bullet}+r_{\mathrm{NO}^\bullet}}\ \ln\left(\frac{2\alpha^{TC}_{\mathrm{O}_2^-{}^\bullet}\ \sigma_{TC}(T)}{\alpha^{EC}_{\mathrm{NO}^\bullet}\ \sigma_{EC}(T)}\right), \tag{27}$$

where ln(·) denotes natural logarithm.

Combining this relation with Eqs.23, 25 and 26, after simplifying we obtain

$$[\mathrm{ONOO}]_{EC}(0,T) \approx$$

$$\frac{\tau_{\mathrm{ONOO}}}{r_{\mathrm{ONOO}}}\left(2\alpha^{TC}_{\mathrm{O}_2^-{}^\bullet}\ \sigma_{TC}(T)\right)^{\frac{r_{\mathrm{O}_2^-{}^\bullet}(r_{\mathrm{ONOO}}-r_{\mathrm{NO}^\bullet})}{(r_{\mathrm{O}_2^-{}^\bullet}+r_{\mathrm{NO}^\bullet})r_{\mathrm{ONOO}}}} \left(\alpha^{EC}_{\mathrm{NO}^\bullet}\ \sigma_{EC}(T)\right)^{\frac{(r_{\mathrm{O}_2^-{}^\bullet}+r_{\mathrm{ONOO}})r_{\mathrm{NO}^\bullet}}{(r_{\mathrm{O}_2^-{}^\bullet}+r_{\mathrm{NO}^\bullet})r_{\mathrm{ONOO}}}}\ \mathrm{e}^{-\frac{(r_{\mathrm{O}_2^-{}^\bullet}+r_{\mathrm{ONOO}})}{(r_{\mathrm{O}_2^-{}^\bullet}+r_{\mathrm{NO}^\bullet})r_{\mathrm{ONOO}}}L_1}\ . \tag{28'}$$

This formula captures the case when the concentrations of superoxide and nitric oxide equal at some point between the cultures of transformed and effector cells, i.e. the case of $0 \leq L_r \leq L_1$. If less superoxide is produced than the amount of nitric oxide released by effector cells that diffuses to transformed cells (so that the 'meeting point' would be $L_r < 0$) or if less nitric oxide is produced than superoxide diffusing to effector cells (so that $L_r > L_1$), Eq. 28' cannot be derived in this form as the underlying assumption that $\sqrt{1+x} - 1 \approx \sqrt{x}$ does not hold in Eqs.25-26. Eq.28' would provide unrealistically high results in such cases. A simple solution to this problem is considering, similarly to the case of peroxynitrite from nitric oxide released by transformed cells discussed above, that not more peroxynitrite can be formed than the levels of superoxide and nitric oxide present at transformed cells:

$$[\mathrm{ONOO}]_{EC}(0,T) \approx$$

$$\frac{\tau_{\mathrm{ONOO}}}{r_{\mathrm{ONOO}}} \min \left\{ \begin{array}{c} \alpha^{TC}_{\mathrm{O}_2^{-\bullet}} \sigma_{TC}(T), \quad \frac{\alpha^{EC}_{\mathrm{NO}^\bullet} \sigma_{EC}(T)}{2} \mathrm{e}^{-\frac{L_1}{r_{\mathrm{NO}^\bullet}}}, \\ \left(2\alpha^{TC}_{\mathrm{O}_2^{-\bullet}} \sigma_{TC}(T)\right)^{\frac{r_{\mathrm{O}_2^{-\bullet}} (r_{\mathrm{ONOO}} - r_{\mathrm{NO}^\bullet})}{(r_{\mathrm{O}_2^{-\bullet}} + r_{\mathrm{NO}^\bullet}) r_{\mathrm{ONOO}}}} \left(\alpha^{EC}_{\mathrm{NO}^\bullet} \sigma_{EC}(T)\right)^{\frac{(r_{\mathrm{O}_2^{-\bullet}} + r_{\mathrm{ONOO}}) r_{\mathrm{NO}^\bullet}}{(r_{\mathrm{O}_2^{-\bullet}} + r_{\mathrm{NO}^\bullet}) r_{\mathrm{ONOO}}}} \mathrm{e}^{-\frac{(r_{\mathrm{O}_2^{-\bullet}} + r_{\mathrm{ONOO}})}{(r_{\mathrm{O}_2^{-\bullet}} + r_{\mathrm{NO}^\bullet}) r_{\mathrm{ONOO}}} L_1} \end{array} \right\}. \quad (28)$$

Note that the first two terms are directly proportional to the densities of transformed and effector cells, respectively. On the other hand, the third term depends on cell densities (and per-cell release rates of superoxide and nitric oxide) in a highly non-trivial manner: Typically nitric oxide is significantly more stable than peroxynitrite, so that $r_{\mathrm{NO}^\bullet} > r_{\mathrm{ONOO}}$, and hence the first exponent is negative. This means that the yields of peroxynitrite at x=0 *decrease* with increasing density of transformed cells or per-cell release of superoxide. The reason for this inversed behaviour is the following: Keeping the production of nitric oxide by effector cells at L_1 constant and increasing the production of superoxide by transformed cells at x=0, the effective reaction point gets farer away from the transformed cells, i.e. L_r increases. More superoxide and nitric oxide are present and hence more peroxynitrite is formed at this new reaction point. However, this effect is outweighed by the increased distance peroxynitrite has to diffuse to reach transformed cells, so that its levels there actually decrease.

1.3.3 Analytical approach: Summary

Taken together, the analytical approach consists in calculating peroxynitrite levels by Eqs.28 and 24c for nitric oxide derived from effector cells and transformed cells, respectively, the concentrations of hypochlorous acid by Eq.21a, and finally by converting these to signalling efficiency in terms of the yields of hydroxyl radicals by Eq.1.

The analytical formulas derived above are based on three assumptions:

(1) Reactions can be approximated by local absorption;
(2) Interplay between the pathways and modes can be neglected;
(3) Diffusion lengths of all species are much smaller than the height L of intercellular medium (the size of the region of interest), distance L_1 between cell cultures, and the distance L_r between the transformed population and the effective source of peroxynitrite.

The assumption (3) has been introduced only to keep the analytical formulas relatively simple; it is equivalent to assuming that the species be relatively short-lived. Full formulas could be derived without this assumption but are not reported here for the sake of simplicity. On the other hand, abandoning assumption (2) of independent pathways leads to sets of coupled equations that cannot be solved analytically. The assumption (1) is crucial for the modelling presented here, although e.g. a reaction-diffusion equation for superoxide with its dismutation only

but without any further reactions could be solved in terms of special functions. Note that the assumption (1) is almost perfectly fulfilled due to the high reaction rate constants in the given signalling cascade, in particular the one for the reaction of superoxide with nitric oxide. The approximation by local absorption, assumption (1), works however surprisingly well (cf. the results presented in the main text) also for superoxide dismutation, whose reaction rate constant is 4 orders of magnitude lower; the short lifetime of superoxide assures this.

1.4 Iterative method

A significant improvement to the above discussed analytical formulas can be obtained if the defining Eqs.15-18 are not solved analytically with numerous approximations as discussed above but iteratively using the perturbation theory:

The method starts from concentrations of superoxide and nitric oxide ($[\mathrm{O_2^{-\bullet}}]^{(0)}$, $[\mathrm{NO^\bullet}]^{(0)}$) that are calculated as if there were no mutual reactions at all. That is, $[\mathrm{O_2^{-\bullet}}]^{(0)}$ is calculated from Eqs.15 where however terms with $[\mathrm{NO^\bullet}]$ are omitted in absorption terms β_0 and β_r. In an analogous way, $[\mathrm{NO^\bullet}]^{(0)}$ is obtained from Eqs.16-17. The effective reaction point $L_r^{(0)}$ is calculated using Eq.18 from these concentration profiles without reactions, as the point where $[\mathrm{O_2^{-\bullet}}]^{(0)}(L_r^{(0)}) = [\mathrm{NO^\bullet}]^{(0)}(L_r^{(0)})$.

In the first iterative step, refined concentrations of superoxide and nitric oxide are calculated that account for first-order effects of reactions. Here, $[\mathrm{O_2^{-\bullet}}]^{(1)}$ is obtained from Eqs.15 where absorption terms $\beta_0^{(0)}$ and $\beta_r^{(0)}$ are calculated using $[\mathrm{NO^\bullet}]^{(0)}$. In an analogous way, $[\mathrm{NO^\bullet}]^{(1)}$ is obtained from Eqs.16-17 with absorption terms containing $[\mathrm{O_2^{-\bullet}}]^{(0)}$. The effective reaction point is refined to $L_r^{(1)}$ given by $[\mathrm{O_2^{-\bullet}}]^{(1)}(L_r^{(1)}) = [\mathrm{NO^\bullet}]^{(1)}(L_r^{(1)})$.

In the second iterative step, $[\mathrm{O_2^{-\bullet}}]^{(2)}$ is obtained from Eqs.15 with absorption terms $\beta_0^{(1)}$ and $\beta_r^{(1)}$ that use $[\mathrm{NO^\bullet}]^{(1)}$; similarly is $[\mathrm{NO^\bullet}]^{(2)}$ calculated, and the effective reaction point is refined by $[\mathrm{O_2^{-\bullet}}]^{(2)}(L_r^{(2)}) = [\mathrm{NO^\bullet}]^{(2)}(L_r^{(2)})$.

In an analogous way the iterative procedure is followed to higher-order terms. In this paper the iterative procedure has been stopped when the concentrations of superoxide and nitric oxide at the effective reaction point L_r differed by less than 0.01 %. If this criterion was not met, indicating a poorly convergent procedure, the procedure was limited to 500 iterations in order to restrict the needed computation time.

Contrary to the analytical formulas, in the iterative procedure the assumption (3) on short-lived signalling species has not been made. Full versions of Eqs.19 derived by Wolfram Mathematica® Version 9 have been used, keeping all factors accounting for the limited distances L, L_1 and L_r such as $1 - \mathrm{e}^{-L/r_{\mathrm{O_2^{-\bullet}}}}$ throughout the calculations. Obviously, not neglecting these factors is especially important when modelling the reaction system for small distances between the populations of transformed and effector cells and/or for low amounts of medium.

2. Results

2.1 Approximating mutual reactions by local absorption

Mutual reactions of signalling species can be approximated by local absorption. This is illustrated in Fig.S2 for the reaction between superoxide and nitric oxide for three values of superoxide release rate per transformed cell, namely 10^{-18} mol/s, 10^{-16} mol/s (the standard value from Tab.3), and 10^{-14} mol/s. Quasi-steady-state concentration profiles obtained by detailed numerical simulations are depicted by histograms. Note that nitric oxide (green lines) has been generated from normal cells (at x=1 mm) as well as from transformed cells (at x=0), whereas only transformed cells produce superoxide (red lines). The iterative procedure reasonably approximates the concentration profile of normal cell-derived nitric oxide from its source at x=1 mm (and, similarly, of superoxide from its source at x=0) down to the effective reaction point L_r (about 0.04, 0.35, and 0.5 mm, respectively, for the three release rates of superoxide). Outside these regions, the iteratively calculated concentration profiles underestimate the effect of reaction; they decrease with the same linear coefficient (extinction coefficient) as if no reaction took place. However, as only species concentrations at $x=L_r$ are needed for the formula that provides the reaction flux in this approximation, the predicted yields and spatially-dependent concentrations of peroxynitrite (blue lines) are reproduced satisfactorily. Even an overestimation of the effective reaction point for the highest release rate of superoxide (Panel C) does not affect peroxynitrite levels at transformed cells (x=0).

Note also that the shift of the effective reaction point L_r farer away from the transformed cells with increasing superoxide release by transformed cells means that more peroxynitrite diffuses to effector cells at x=1 mm (Fig.S2 Panels A-C). If the resulting $^{\bullet}$OH attacks to effector cells are sufficient to trigger their apoptosis, the selectivity of IIA to the transformed phenotype is reduced. The iterative approach may overestimate this effect (Fig.S2 Panel C).

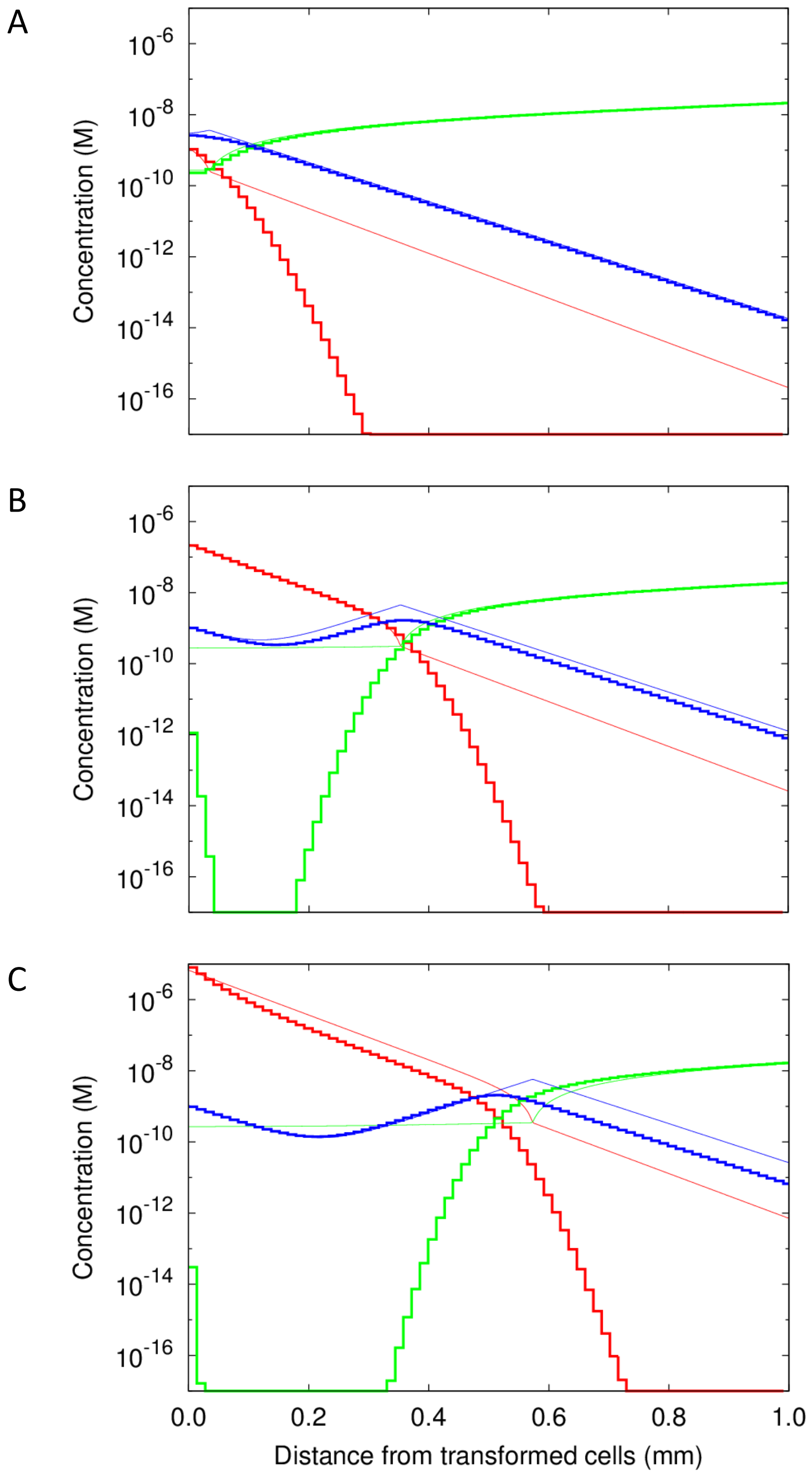

Figure S2: Mutual reactions between signaling species can be approximated by local absorption terms. Quasi-steady-state concentration profiles of superoxide (red), nitric oxide (green) and their reaction product, peroxynitrite (blue) obtained by numerical simulations (thick histograms) and their approximation by the iterative procedure (thin lines). Shown are results for superoxide release rates of 10^{-18} mol/s (Panel A), 10^{-16} mol/s (the standard value from Tab.3 in the main text; Panel B), and 10^{-14} mol/s (Panel C) per transformed cell, with other parameters taking their standard values.

2.2 Roles of parameters affecting the signalling efficiency

The effectiveness of IIA signalling, assessed in terms of the yields of hydroxyl radicals at transformed cells, depends sigmoidally on the release rate of $NO^\bullet$ by effector cells (Fig.S3 Panel A). As soon as this rate exceeds about 2×10^{-17} mol s^{-1} per cell (for other parameters taking their standard values from Tab.3), the peroxynitrite pathway with $NO^\bullet$ derived from effector cells (green line) dominates the signalling. Analytical and iterative calculations (dashed and solid blue lines) correctly reproduce the maximal signalling efficiency effectiveness but overestimate the effectiveness of high $NO^\bullet$ releases; this is due to having neglected the autoxidation of $NO^\bullet$, reactions #9-11 in Table 1, as demonstrated by the results of numerical simulations neglecting these reactions (empty squares).

Simulations with the standard parameters are insensitive to the lifetime of $NO^\bullet$ (Fig.S3 Panel B), as this parameter does not critically influence the peroxynitrite pathway in its autocrine mode (cf. Eq.24c) and the inter-culture mode is too weak (green line). However, at higher release rates of $NO^\bullet$ from effector cells where the peroxynitrite pathway in its inter-culture mode plays an important role, an increase in the lifetime of $NO^\bullet$ does enhance the signalling effectiveness (simulation results shown by empty symbols); again, the iterative approach (dash-dotted blue line) overestimates the simulation results due to having neglected the autoxidation of $NO^\bullet$.

Similar overall sigmoid behaviour is obtained for varying the level of peroxidase present (Fig.S3 Panel C) and the lifetime of HOCl (Fig.S3 Panel D). The outcome of the signalling is in the studied ranges only mildly affected by the lifetimes of hydrogen peroxide or peroxynitrite (Fig.S3 Panels E-F). Geometrical factors such as the distance between the cultures or the height of cell culture medium influence the signalling too (Fig.S3 Panels G-H). The analytical formulas provide correct trends, except for the role of geometrical factors, due to having neglected in the formulas factors such as $1\text{-}\exp(-L/r_{NO})$. The iterative approach accounts for these factors, and reproduces the simulation results correctly. In fact, the results of the iterative approach are systematically slightly higher than the simulation results. This issue, however, seems to be related to a slight inaccuracy of the numerical simulations, as indicated in Fig.S3 Panel G by the results of exemplary numerical simulations using twice smaller spatial grid (red points) or time steps (yellow points). Due to their high computational expensiveness and only minor differences in the results, such simulations with finer steps have been limited to the few cases presented here.

The results of simulations and modelling on the effect of antioxidants such as superoxide dismutase (SOD) that convert superoxide into hydrogen peroxide are presented in Fig.S3 Panels I-J. In Panel I, increasing levels of SOD have been considered in addition to the standard rate of superoxide removal, described by its lifetime of 1.7 s. The simulation results (points) are nicely reproduced by the iterative approach (solid blue line), with a slight overestimation at high SOD levels; the analytical approach (dashed blue line) shows somewhat larger deviations from the numerical simulations. Note that at low and medium SOD levels the HOCl pathway (red line) dominates, while at high SOD levels the major contribution to IIA signalling comes from the peroxynitrite pathway with $NO^\bullet$ derived from effector cells (green line). The shapes and mutual positions of these efficiency curves for individual signalling modes vary with system parameters. Enhancing the lifetimes of superoxide and nitric oxide and reducing the level of POD separates the two peaks and forms a local minimum in overall IIA signalling efficiency at SOD concentration of about 2×10^{-10} M (Fig.S3 Panel J).

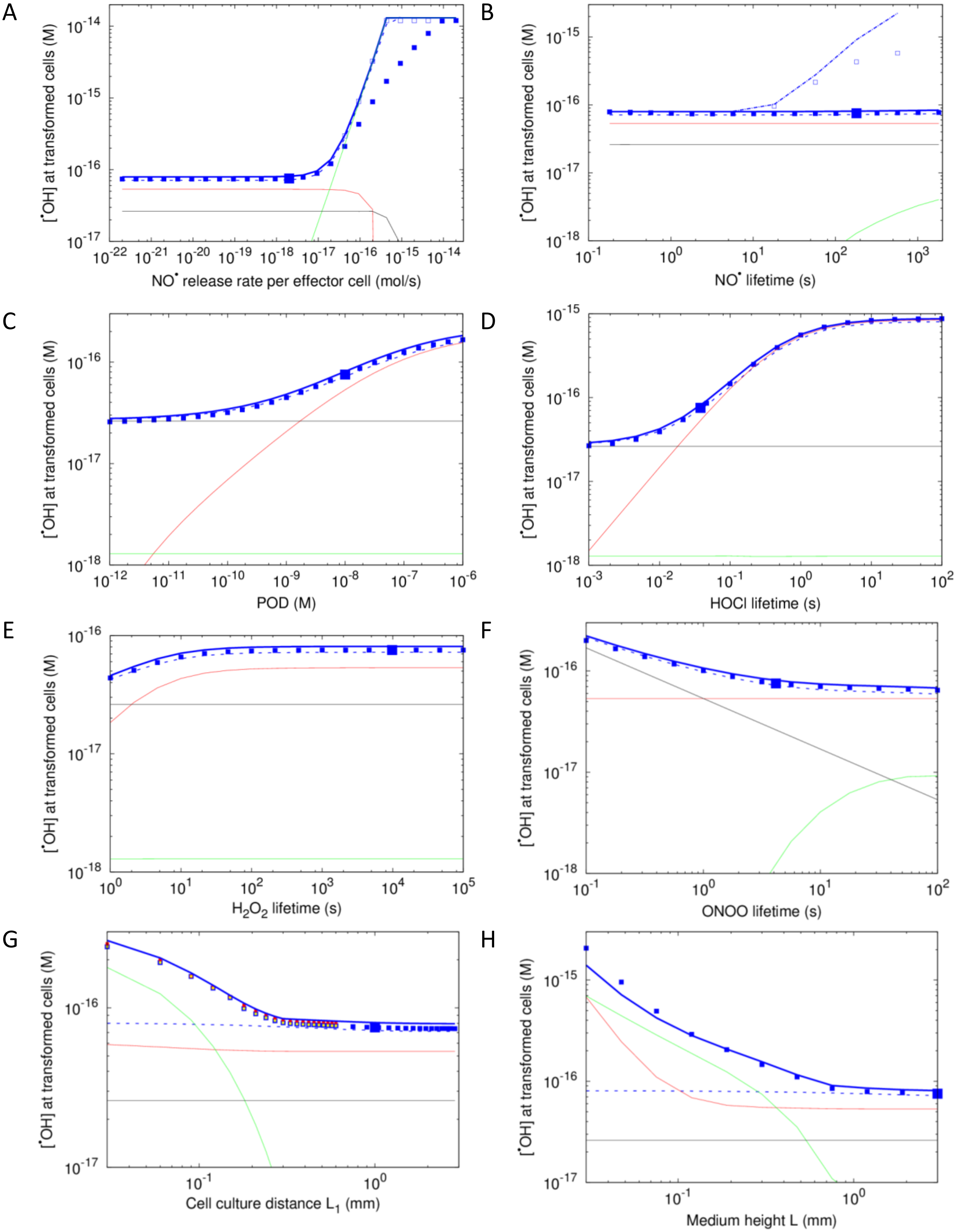
A
B
C
D
E
F
G
H
[•OH] at transformed cells (M)
NO• release rate per effector cell (mol/s)
NO• lifetime (s)
POD (M)
HOCl lifetime (s)
H2O2 lifetime (s)
ONOO lifetime (s)
Cell culture distance L1 (mm)
Medium height L (mm)

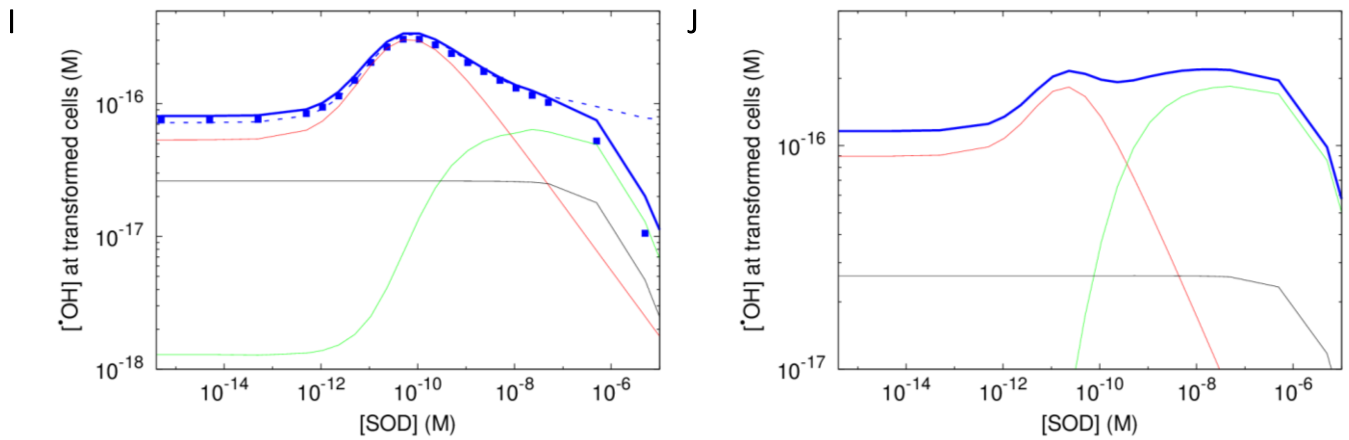

Figure S3: Effectiveness of IIA signaling in dependence on the release rate of NO• by effector cells (Panel A), lifetime of NO• (Panel B), the amount of peroxidase present (Panel C), lifetimes of HOCl (Panel D), H_2O_2 (Panel E) and peroxynitrite (Panel F), geometrical setup (Panels G-H), and the level of superoxide dismutase, SOD (Panels I-J). The results of numerical simulations are depicted by blue symbols; large symbols correspond to standard parameters from Tab.3 in the main text. Lines show the results of the iterative approach (solid blue lines), analytical formulas (dashed blue lines), and the contributions from the peroxidase pathway (red lines) and from the peroxynitrite pathway in autocrine (black lines) and inter-culture modes (green lines). In Panel A, empty symbols depict simulations without the autoxidation of NO•, reactions #9-11 in Table 1. In Panel B, empty symbols and dash-dotted blue line capture simulations and iterative results with NO• release rate per effector cell enhanced to 9×10^{-17} mol/s. In Panel G, the cell culture distance L_1 has been varied, keeping the height of the cell culture medium at L = 3 mm. Also presented are results of simulations with twice smaller spatial grid (red symbols) or twice smaller time steps (yellow symbols). In Panel H, the height of the culture medium has been varied, keeping the inter-culture distance L_1 = L/3. In Panel I, in addition to the non-specific removal of superoxide with the standard lifetime of 1.7 s, its conversion to H_2O_2 by SOD has been considered. In Panel J, results of exemplary calculations with the iterative approach are shown with lifetimes of superoxide and nitric oxide increased three and ten times and peroxidase level reduced ten times, other parameters unchanged.

2.3 Roles of basic cellular parameters

In Figure S4 is shown the effect of varying model parameters that describe basic cellular properties. Allowing transformed cells to reach higher densities somewhat enhances the percentage of apoptotic cells in the first maximum but leaves the long-term percentage largely unaffected (Figure S4A). On the contrary, allowing normal cells to grow to higher densities enhances the available nitric oxide and peroxidase and makes virtually all transformed cells undergo apoptosis (Figure S4B). Reducing the rate of removal of apoptotic cells, i.e. increasing the characteristic time t_{rm} of this process, leaves more apoptotic cells present and, hence, increases their percentage (Figure S4C). The rate and extent of apoptosis in the transformed population also increase if cellular repair of membrane damage gets compromised, i.e. if characteristic time for repair t_{rep} increases (Figure S4D). Enhancing cellular sensitivity to membrane damage, i.e. reducing the amount of damage that triggers apoptosis, shifts the onset of apoptosis towards earlier times and enlarges its overall extent (Figure S4E). Increasing the slope of the non-linear cell response to membrane damage also affects the rate and extent of apoptosis; it may make the kinetics of apoptosis quickly oscillating (Figure S4F). Varying the characteristic duration of apoptosis execution naturally also modulates the kinetics of apoptosis manifestation (Figure S4G). Faster proliferation of transformed or effector cells affect apoptosis as shown in Figure S4H-I. The characteristic time describing spontaneous induction of apoptosis influences the results only mildly (Figure S4J).

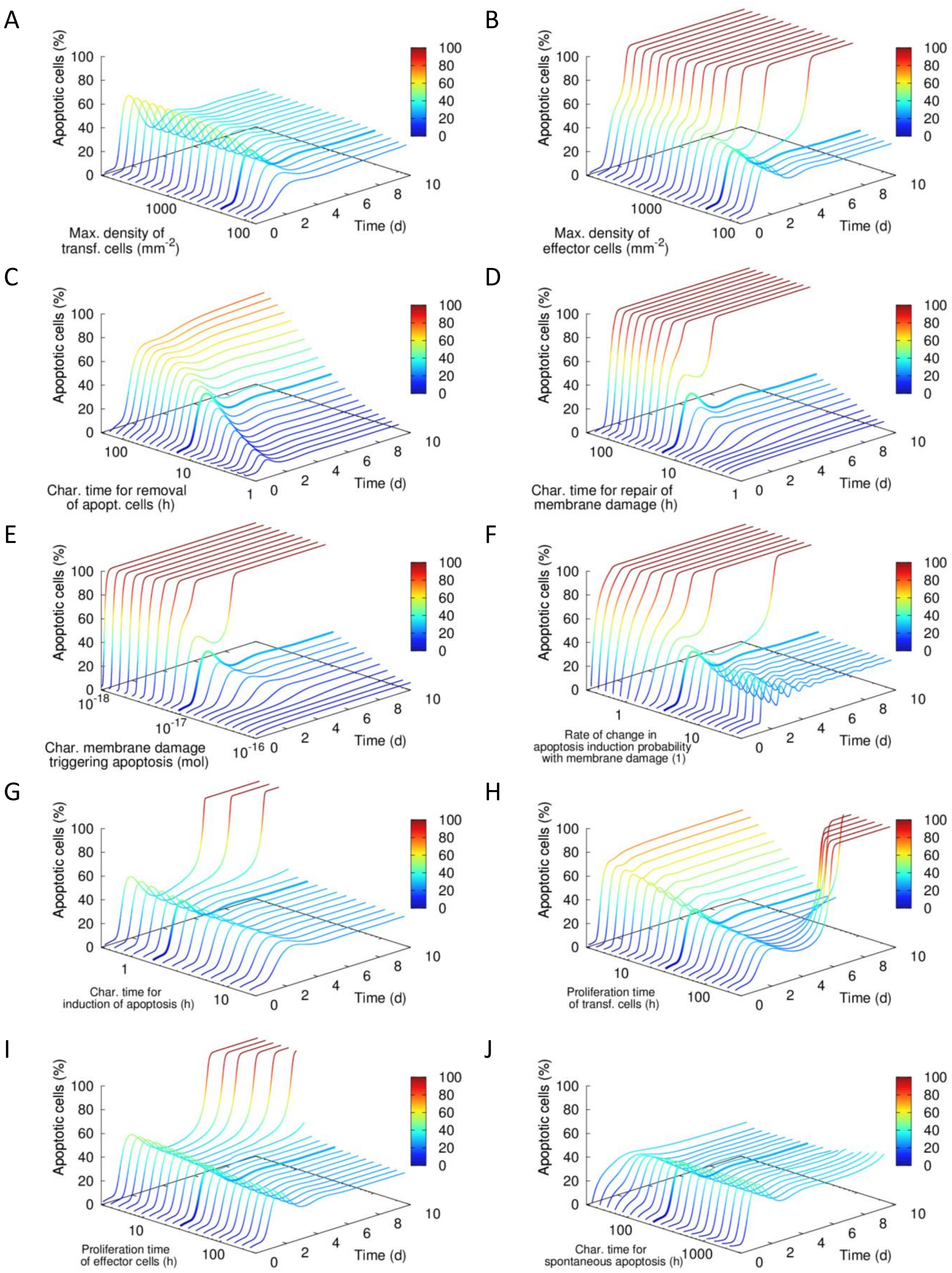

Figure S4: Influence of basic cell parameters on the percentage of apoptosis scored. The results for standard parameters (Tab.2 in the main text) are highlighted by the thick curves.

References

Alberts B, Johnson A, Lewis J, Raff M, Roberts K, Walter P (2002). Molecular Biology of the Cell (4th edition), Chapter 10 Membrane Structure - The Lipid Bilayer. New York: Garland Science; 2002. ISBN: 0-8153-3218-1, ISBN: 0-8153-4072-9

Bechtel W, Bauer G. Catalase protects tumor cells from apoptosis induction by intercellular ROS signaling. Anticancer Res. 2009 Nov;29(11):4541-57.

Deborde M, von Gunten U. Reactions of chlorine with inorganic and organic compounds during water treatment-Kinetics and mechanisms: a critical review. Water Res. 2008 Jan;42(1-2):13-51.

Engelmann I, Dormann S, Saran M, Bauer G. Transformed target cell-derived superoxide anions drive apoptosis induction by myeloperoxidase. Redox Rep. 2000;5(4):207-14.

Gray B, Carmichael AJ. Kinetics of superoxide scavenging by dismutase enzymes and manganese mimics determined by electron spin resonance. Biochem J. 1992;281 (Pt 3):795-802.

Kundrát P, Bauer G, Jacob P, Friedland W (2012). Mechanistic modelling suggests that the size of preneoplastic lesions is limited by intercellular induction of apoptosis in oncogenically transformed cells. Carcinogenesis. 2012 Feb;33(2):253-9. doi: 10.1093/carcin/bgr227.

Lobachev V L, Rudakov E S (2006). The chemistry of peroxynitrite. Reaction mechanisms and kinetics. Russ Chem Rev 2006;75:375-96.

Portess D. An investigation into cellular signalling leading to the selective induction of apoptosis in transformed cells following radiation. D.Phil. Thesis, University of Oxford, 2007

Radi R, Beckman JS, Bush KM, Freeman BA (1991). Peroxynitrite-induced membrane lipid peroxidation: the cytotoxic potential of superoxide and nitric oxide. Arch Biochem Biophys 1991;288:481-7.

Saran M, Bors W. Signalling by O2-. and NO.: how far can either radical, or any specific reaction product, transmit a message under in vivo conditions? Chem Biol Interact. 1994;90(1):35-45
